# The impact of interventions to promote healthier ready‐to‐eat meals (to eat in, to take away or to be delivered) sold by specific food outlets open to the general public: a systematic review

**DOI:** 10.1111/obr.12479

**Published:** 2016-11-29

**Authors:** F. C. Hillier‐Brown, C. D. Summerbell, H. J. Moore, A. Routen, A. A. Lake, J. Adams, M. White, V. Araujo‐Soares, C. Abraham, A. J. Adamson, T. J. Brown

**Affiliations:** ^1^ Obesity Related Behaviours Research Group, School of Medicine, Pharmacy and HealthDurham UniversityStockton‐on‐TeesUK; ^2^ Fuse – UKCRC Centre for Translational Research in Public HealthNewcastle Upon TyneUK; ^3^ School of Sport Exercise and Health SciencesLoughborough UniversityLoughboroughUK; ^4^ Centre for Public Policy & Health, School of Medicine, Pharmacy & HealthDurham UniversityStockton‐on‐TeesUK; ^5^ UKCRC Centre for Diet and Activity Research (CEDAR), MRC Epidemiology UnitUniversity of CambridgeCambridgeUK; ^6^ Institute of Health & SocietyNewcastle UniversityNewcastle Upon TyneUK; ^7^ Psychology Applied to HeathUniversity of Exeter Medical School, University of ExeterExeterUK; ^8^ Human Nutrition Research CentreNewcastle UniversityNewcastle Upon TyneUK

**Keywords:** Diet, food environments, ready‐to‐eat meals, restaurants, systematic review, takeaways

## Abstract

**Introduction:**

Ready‐to‐eat meals sold by food outlets that are accessible to the general public are an important target for public health intervention. We conducted a systematic review to assess the impact of such interventions.

**Methods:**

Studies of any design and duration that included any consumer‐level or food‐outlet‐level before‐and‐after data were included.

**Results:**

Thirty studies describing 34 interventions were categorized by type and coded against the Nuffield intervention ladder: *restrict choice* = trans fat law (*n* = 1), changing pre‐packed children's meal content (*n* = 1) and food outlet award schemes (*n* = 2); *guide choice* = price increases for unhealthier choices (*n* = 1), incentive (contingent reward) (*n* = 1) and price decreases for healthier choices (*n* = 2); *enable choice* = signposting (highlighting healthier/unhealthier options) (*n* = 10) and telemarketing (offering support for the provision of healthier options to businesses via telephone) (*n* = 2); and *provide information* = calorie labelling law (*n* = 12), voluntary nutrient labelling (*n* = 1) and personalized receipts (*n* = 1). Most interventions were aimed at adults in US fast food chains and assessed customer‐level outcomes. More ‘intrusive’ interventions that restricted or guided choice generally showed a positive impact on food‐outlet‐level and customer‐level outcomes. However, interventions that simply provided information or enabled choice had a negligible impact.

**Conclusion:**

Interventions to promote healthier ready‐to‐eat meals sold by food outlets should restrict choice or guide choice through incentives/disincentives. Public health policies and practice that simply involve providing information are unlikely to be effective.

## Background

Ready‐to‐eat meals (to eat in, to take away or to be delivered) sold by specific food outlets that sell ready‐to‐eat meals as their main business are often more energy dense and nutrient poor compared with meals prepared and eaten at home 1. Furthermore, the consumption of these ready‐to‐eat meals is associated with higher energy and fat and lower micronutrient intake 2. Eating takeaway or fast food is associated with excess weight gain and obesity 3, 4.

The popularity and availability of ready‐to‐eat meals have risen considerably over the last few decades in many high‐income and middle‐income countries 5, 6, 7. For example, around one‐fifth to one‐quarter of the UK population eat takeaway meals at home at least once per week 7. There is some evidence that food outlets selling takeaway meals and fast foods are clustered in areas of socioeconomic deprivation 8. Ready‐to‐eat meals sold by food outlets, particularly in deprived areas, are therefore an important target for public health intervention 9.

In some countries, national and local government health departments have worked with national and regional food outlet chains to promote healthier ready‐to‐eat meals. Many of these interventions have used ‘health by stealth’ approaches, such as reformulation (particularly salt reduction, the removal of trans fats and energy reductions) and removal of condiments from tables in sit‐in eateries. Other interventions have focused on promoting smaller portion sizes and providing consumers with better nutritional information (e.g. calorie labelling on menus) 10.

Bowen *et al*. 11 recently completed a critical literature review, guided by a socioecological framework, on the effects of different types of environmental and policy interventions on healthy eating, from a US perspective. They concluded that, whilst the evidence reviewed did not support menu labelling as an effective strategy to change purchasing patterns, additional strategies to enhance menu labelling practices, and strategies beyond labelling (including implementation of nutritional standards), may be useful. The authors concluded that this literature requires further review.

The aim of this evidence synthesis was therefore to systematically review the international literature on the impact 1
*Impact* in this paper is used to describe a change in an outcome of interest associated with an intervention. In uncontrolled before‐and‐after (or pre/post) studies, impact was assessed as the change in the outcome of interest from baseline to post intervention. In randomized controlled trials (RCTs) and non‐RCTs, impact was assessed as the difference in change in the outcome of interest in the intervention group compared with the controls. Of note, where we report impact, we do so alongside the methodological quality of the study (strong, moderate or weak); studies without a control could only achieve a quality assessment of moderate or weak. We appreciate that impact results from uncontrolled studies should be treated with caution (e.g. http://handbook.cochrane.org/chapter_21/21_4_assessment_of_study_quality_and_risk_of_bias.htm). The absence of a comparison group makes it impossible to know what would have happened without the intervention. Some of the particular problems with interpreting data from uncontrolled studies include susceptibility to problems with confounding (including seasonality) and regression to the mean. of interventions to promote healthier ready‐to‐eat meals (to eat in, to take away or to be delivered) sold by specific food outlets accessible to the general public.

For the purposes of this review, we have defined ready‐to‐eat meals as complete meals that need no further preparation and are bought from food outlets, to eat in, to take away or to be delivered. For example, a bought sandwich or salad box would be included in this definition. However, a packet of crisps/potato chips and a drink, or a chocolate bar, would not be considered a ready‐to‐eat meal, even if the person consuming them was doing so in replacement of a meal. We acknowledge that terminology in this field is challenging. The literature in this field often includes references to ‘takeaways’, ‘fast food’ and ‘out of home eating’. In the USA, the term ‘takeout meals’ is often used, and in Australia, they speak of ‘meals prepared outside the home’. In the absence of a globally agreed definition, we have used the term ‘ready‐to‐eat meals’ throughout, and it includes ‘takeaways’, ‘fast food’, ‘out of home eating’, ‘takeout meals’ and ‘meals prepared outside the home’.

## Methods

The systematic review was undertaken using established methods based on those used by the National Institute for Health and Care Excellence 12, and the findings are reported according to the Preferred Reporting Items for Systematic Reviews and Meta‐Analyses guidelines 13. The review is registered with the International Prospective Register of Systematic Reviews (PROSPERO) (registration no. CRD42013006931), and the protocol is published 14.

### Inclusion criteria

#### Setting

The specific food outlets we included were those that, as their main business, sold ready‐to‐eat meals and were openly accessible to the general public. Supermarkets and general food stores selling ready‐to‐eat meals (e.g. salad boxes and sandwiches) were not included, but cafes and restaurants within supermarkets and other retail stores selling ready‐to‐eat meals were. Food outlets that provided ready‐to‐eat meals free of charge (e.g. community‐based lunch clubs for the elderly or homeless) were excluded. We also excluded food outlets that are not openly accessible to the general public, including those based in schools, universities, workplaces and health/social care institutions. This was for two reasons: first, the effects of interventions to promote the sale of healthier meals in these environments have previously been reviewed 15, 16, 17. Second, the relationship between the provider (e.g. on behalf of the education authority or employer) and consumer (e.g. student or employee) of ready‐to‐eat meals in these institutions is somewhat different to that between a business and the general public (e.g. the meals may be subsidized).

#### Interventions

Any type of intervention that aimed to change the practices of food outlets in order to promote healthier menu offerings was included. Interventions identified for review were assessed for type of intervention; 11 categories were identified. Box 1 describes each type of intervention category as defined by the review team, and for convenience, they are ordered by where they sit on the Nuffield ladder 18 (described in the following). Interventions that were categorized as ‘signposting’‐type studies were defined as those that highlighted to customers the healthier, or less healthy, menu options available. This was usually carried out using symbols next to menu items, but table signage and posters were other methods used. Signposting differs from calorie labelling on menus as it provides some indication of the ‘healthfulness’ of a menu item rather than just providing information. Interventions that were categorized as ‘telemarketing of healthy food choices’‐type studies were defined as those that involved a phone‐based direct marketing strategy and a variety of free services offered to businesses including menu guidelines for the provision of healthy choices.

**Box 1. Summary description of the intervention categories**

**Table:** 

Intervention category and description of interventions identified by review	Nuffield intervention ladder definition^a^
*Trans fat law*: Restriction of all food service establishments, including both chain and non‐chain food outlets, from using, storing or serving food that contains partially hydrogenated vegetable oil and has a total of 0.5 g or more trans fat per serving	Restrict choice
*Changing pre‐packed children's meal content*: Pre‐packed meal content changed to include healthier options, smaller portion sizes of less healthy options and/or removal of other less healthy options	Restrict choice
*Food outlet award schemes*: Interventions that include an assessment of food outlet practice(s) using predefined criteria, together with some sort of accreditation if the food outlet met the criteria	Restrict choice (variable depending on scheme, but those included in this review were all categorized as restrict choice)
*Price increases for unhealthier choices*: Price increase applied to less healthy menu options	Guide choice (disincentives)
*Incentive (contingent reward)*: A conditional reward is provided only after the target behaviour (e.g. choice of a healthier option) is performed	Guide choice (incentives)
*Price reductions for healthier choices*: Price reduction applied to healthier menu options	Guide choice (incentives)
*Signposting*: Interventions that highlighted to customers the healthier, or less healthy, menu options available	Enable choice
*Telemarketing of healthy food choices*: Phone‐based direct marketing strategy; variety of free services offered to businesses including menu guidelines for the provision of healthy choices	Enable choice
*Calorie labelling law*: Mandatory posting of calorie values of each option on menus in chain food outlets	Provide information
*Voluntary calorie labelling*: Voluntary posting of calorie values of each option on menus in chain food outlets	Provide information
*Personalized receipts*: Receipts that included personalized suggestions designed to reduce fat and calorie consumption	Provide information

a Definition from the Nuffield ladder 18 starting with the most intrusive; eliminate choice, restrict choice, guide choice (disincentives), guide choice (incentives), guide choice (default policy), enable choice, provide information, do nothing.

#### Outcomes

Any outcome that included consumer or food outlet outcomes is included. Consumer outcomes could include dietary outcomes (e.g. energy intake), purchasing behaviour (e.g. sales data) and attitudes towards healthier menu choice and preferences. Food outlet outcomes could include changes in retail practices, process outcomes and profit.

#### Study design

A scoping search of the literature, which we conducted in advance of writing the protocol 14, estimated that there would be insufficient evidence from randomized controlled trials to allow us to answer our research question. However, those working in public health policy and practice need to know how best to improve the nutritional quality of ready‐to‐eat meals sold by food outlets. Thus, we took an overarching approach that is used by the National Institute for Health and Care Excellence 12 to identify the best available evidence. Thus, studies of any design that reported outcomes at least once before and once after intervention were included (also called uncontrolled before‐and‐after studies). Studies with and without comparators were included without restriction on the type of comparator.

### Search

Searches identified studies published from January 1993 to October 2015 in the following databases (and interfaces): ASSIA (ProQuest), CINAHL (EBSCOhost), Embase (Ovid), MEDLINE (Ovid), NHS EED (Wiley Cochrane) and PsycINFO (EBSCOhost). Searches were limited to articles written in English. Topic experts were contacted for information about any additional relevant interventions not identified by the electronic search. Key reviews 19, 20, 21 were searched as well as reference lists of included studies. Details of the search strategies can be found in Table S1.

Initial screening of titles and abstracts was conducted by one reviewer (F. H. B.) with a random 10% of the sample independently screened by a second reviewer (H. M.). Agreement between the reviewers was fair (kappa = 0.50) as a result of the second reviewer being more inclusive than the main reviewer. Disagreements between the reviewers were resolved through discussion, and it was agreed that studies initially excluded by the main reviewer and included by the second reviewer were excluded at this stage. Full‐text articles of potentially relevant studies were independently appraised by two researchers (F. H. B. and C. S.). Agreement between the reviewers at this stage was excellent (kappa = 0.80). Any disagreements between reviewers were resolved by discussion.

### Data extraction and quality assessment

Data extraction and quality assessment were conducted independently by two reviewers (all authors contributed), and any discrepancies between reviewers were resolved through discussion with a third reviewer (T. B.). Data were extracted on study characteristics, intervention type and outcomes. Study quality was assessed using the Effective Public Health Practice Project Quality Assessment Tool for Quantitative Studies 22 as recommended by the Cochrane Public Health Review Group 23. This was adapted for the purposes of this review, specifically in terms of the classification of study designs (Table 1).

**Table 1:** Adapted typology of study designs and quality

Study design	Study design quality score
Repeat cross‐sectional	Weak
Repeat cross‐sectional with control	Moderate
Repeat cross‐sectional with cohort subgroup	Moderate
Cohort	Moderate
Repeat cross‐sectional with control and controlled cohort subgroup	Strong
Controlled before–after (same participants)	Strong
Controlled trial	Strong

Data on implementation, including context, collaboration, fidelity, sustainability and differential effects by population demographics (using the PROGRESS [place of residence, race/ethnicity/culture/language, occupation, gender/sex, religion, education, socioeconomic status (SES) and social capital] framework 24), were extracted, using a checklist for obesity‐related interventions 25 adapted from workplace interventions 26. An implementation score (0–10) was assigned based on the number of categories information was reported for. Any cost‐effectiveness data were also extracted.

Data were extracted on the theoretical framework or behavioural model or strategy underpinning each intervention. Interventions were coded according to the Nuffield intervention ladder in order to categorize the interventions in terms of their ‘intrusiveness’ and impingement on personal autonomy 18. We note that the Nuffield ladder uses the term ‘incentive’ loosely. Incentive has been technically defined to mean a reward contingent on changing behaviour, which can be distinguished from a simple price increase or decrease 27, 28. We have made these distinctions explicit in our intervention categories. Interventions were also coded in terms of intervention function and policy category using the Behaviour Change Wheel 29.

### Data synthesis

Given heterogeneity in study designs, intervention types and outcome measures, the results are presented as a narrative synthesis following the Economic and Social Research Council Narrative Synthesis Guidance 30. A ‘summary impact’ of each study was reported (denoted by an arrow), alongside the global rating of study quality (strong, moderate or weak). Studies were classed as ‘effective’ (↑), ‘equally effective’ as the comparison group (↔), ‘effectiveness mixed’ by outcome or gender (↕) or ‘not effective’ (↓). Studies without a control could only achieve a global quality of moderate or weak. Impact was based on change in mean energy purchased where possible (where a decrease in mean energy purchased signified a successful outcome of the intervention, denoted as ↑). Where energy purchased was not reported, impact was based on the primary outcome of the study (e.g. trans fat content of meal, healthy food purchases, catering practices, health promotion practices or menu items available). Impact was assessed using the overall effect for the whole study sample and not by subgroup. Studies with a control group were assessed on change in outcomes between groups at follow‐up; studies without a control group were assessed on change in outcomes from baseline to follow‐up.

## Results

**Figure 1:**
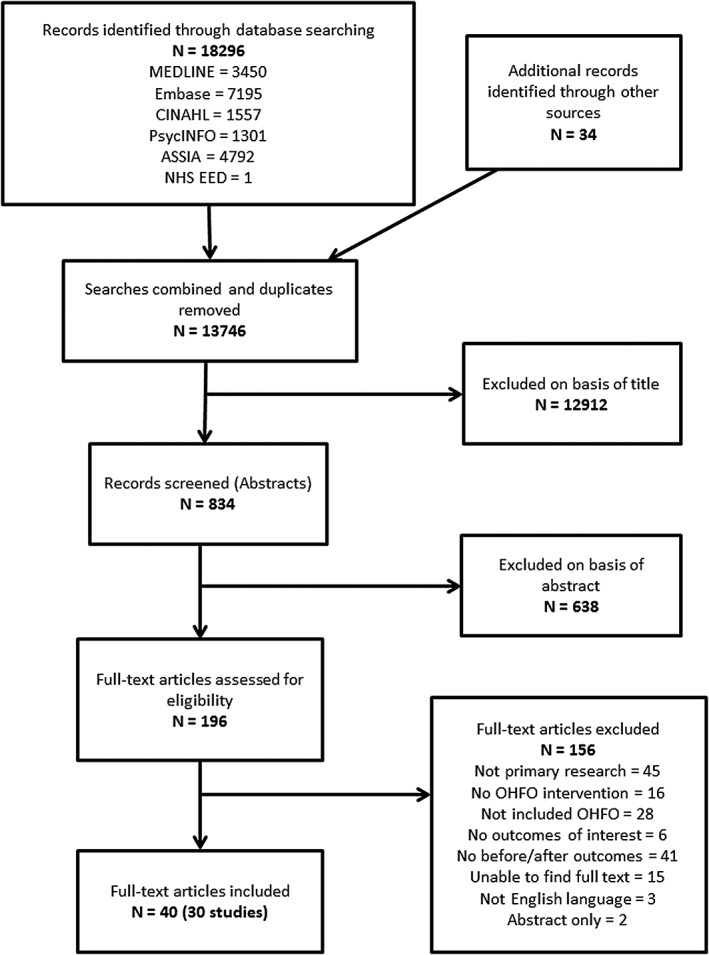
Preferred Reporting Items for Systematic Reviews and Meta‐Analyses flowchart

A total of 30 studies (reported in 40 articles), describing 34 interventions, were included; study flow is reported in a Preferred Reporting Items for Systematic Reviews and Meta‐Analyses flowchart (Fig. 1). Table S1 provides a list of included references. Details of studies that were excluded on screening full‐text articles are listed in Table S2.

### Characteristics of included studies

Study characteristics are summarized in Table S3. Of the 30 included studies, 19 were repeat cross‐sectional studies, 7 with a comparison control group 31, 32, 33, 34, 35, 36, 37 and 12 without 38, 39, 40, 41, 42, 43, 44, 45, 46, 47, 48, 49. These studies were classified as cross‐sectional because the outcomes of the study were mainly measured at the consumer level, so although the same food outlets were assessed at each time point, the customers were most likely to be different. In three of these studies 33, 44, 49, there were subgroup cohorts of customers nested within the repeat cross‐sectional data. Five studies 50, 51, 52, 53, 54 were classified as cohort studies. Two studies were controlled before‐and‐after studies that reported outcomes in the same customers 55 or at the food outlet level in the same food outlets at baseline and follow‐up 56, and four studies were controlled trials 57, 58, 59, 60.

Twenty‐seven of the 30 included studies were based in the USA, two studies were based in Australia 44, 49 and one was based in the UK 50. Twenty‐two studies reported outcomes for adults, three reported outcomes for parents and their children 37, 55, 61 and one study reported child outcomes only 48. For the four remaining studies, food outlets, rather than individuals, were the unit of observation and analysis. Study populations ranged from lower 34 to higher SES 31, 41, 55, 58 and more ethnically diverse samples 57 to mainly Caucasian samples 39, 43, 45. Some studies targeted specific ethnic groups, including Mexican–Americans 53, low‐income African–Americans 59 and low‐income Latino–Americans 46. Many of the studies did not report on population characteristics in detail.

In terms of the types of food outlets targeted, 18 studies focused on chain food outlets and 12 studies were set in other types of food outlet, including three studies in non‐chain food outlets 45, 47, 60; one study each in takeaway food outlets 59; a delicatessen‐style food outlet 58; privately owned fast‐food‐style Mexican food outlets 53; community food outlets that included both counter and table service 42; Latino family‐owned food outlets 46; licensed retail food outlets 52; licensed hotels, clubs and nightclubs 49; restaurants and cafes 44; and small independent catering outlets 50. Most of the chain food outlets were fast food counter service, but other food outlet types included table service or takeaway only. One study was set in food service areas of a large discount department store 41.

Study samples of food outlets varied greatly in size; e.g. one study included just one outlet 58, and another included over 300 31. Study duration ranged from minutes 54 to 7 years 37, and data points ranged from two time points 34 to weekly purchase information for a 125‐week period 32.

Only four studies were assigned a global quality rating of ‘strong’, 10 were rated as ‘moderate’ and 16 were rated ‘weak’ (Table S4). In terms of implementation, scores ranged from 3 to 9 (Table S6). Papers that described the study intervention in detail were more likely to score higher for implementation; however, low scores were not necessarily an indication of poor reporting, just that a number of organizational and implementation factors were not used or explored for the intervention (e.g. theoretical underpinning, collaborative approaches to development and delivery, fidelity of intervention delivery and stakeholder support).

Tables 2a (for studies with customer level outcomes) and 2b (for studies with food‐outlet‐level outcomes) summarize the design, intervention type, context and results for the included studies. Where a study included more than one intervention arm, the results for each have been reported separately (often in different intervention types). Some of the interventions focused on changing customer behaviour directly (e.g. signposting) and some on changing outlet behaviour in an attempt to change customer behaviour (e.g. awards). For more detailed information on study interventions, see Table S5, and for study results, see Table S5.

**Table 2a:** Summary of included studies with customer‐level outcomes (*n* = 23)

Study ID	Study design	Food outlet type	Nuffield intervention ladder	Intervention function	Policy category	Implementation score*	Summary impact (↓↑↔↕) (global quality assessment score)†
Trans fat law (*n* = 1)							
Angell 2012 38, ‡	Repeat cross‐sectional	11 fast food chains, New York City, USA	Restrict choice	Environmental restructuring	Environmental/social planning; legislation	5	↑ (moderate)
Changing pre‐packed children's meal content (*n* = 1)							
Wansink 2014 48	Repeat cross‐sectional	McDonald's restaurants (fast food chain), USA	Restrict choice	Environmental restructuring	Environmental/social planning; communication/marketing	3	↑ (weak)
Price increases for unhealthier choices (*n* = 2)							
Price increases for unhealthier choices only							
Shah 2014 60 (sin tax menu arm)	Controlled clinical trial	One moderately priced restaurant, which specialized in ‘small plates’ to share, USA	Guide choice (disincentives)	Coercion	Fiscal	5	↓ (strong) unhealthy items ordered by men and women
Price increases for unhealthy choices + signposting							
Shah 2014 60 (unhealthy label + sin tax menu arm)	Controlled clinical trial	One moderately priced restaurant, which specialized in ‘small plates’ to share, USA	Guide choice (disincentives)	Environmental restructuring; education; coercion	Communication/marketing; environmental/social planning; fiscal	5	↑ (strong) decrease in unhealthy items ordered by men and women
Incentives (contingent rewards) (*n* = 1)							
Reimann 2015 54	Cohort	Chain sandwich restaurant, USA	Guide choice (incentives)	Incentives	Unclear (customers offered half portions for same price as full portion, plus a lottery ticket)	7	↑ (moderate) customers choosing half‐sized portions
Price reductions for healthier choices (*n* = 2)							
Price reduction for healthier choices only							
Horgen and Brownell 2002 58	Controlled clinical trial	Delicatessen‐style restaurant (cafeteria), USA	Guide choice (incentives)	Incentives	Fiscal	6	↑ (weak) healthy food purchase
Price reduction for healthier choices + health promotion							
Horgen and Brownell 2002 58	Controlled clinical trial	Delicatessen‐style restaurant (cafeteria), USA	Guide choice (incentives)	Environmental restructuring; education; incentives; persuasion; enablement	Communication/marketing; environmental/social planning; fiscal	6	↑ (weak) healthy food purchase
Signposting (*n* = 8)							
Signposting only							
Shah 2014 60 (unhealthy label menu arm)	Controlled clinical trial	One moderately priced restaurant, which specialized in ‘small plates’ to share, USA	Enable choice	Environmental restructuring; education	Communication/marketing; environmental/social planning;	5	↕ (strong) decrease in unhealthy items ordered
Eldridge 1997 41	Repeat cross‐sectional	Food service areas of large discount department store chain, USA	Enable choice	Environmental restructuring; education	Communication/marketing; environmental/social planning	6	↕ (weak) sales of ‘healthier’ food items
Pandya 2013 46	Repeat cross‐sectional	Latino family‐owned restaurants, KS, USA	Enable choice	Environmental restructuring; education	Communication/marketing; environmental/social planning	7	↓ (weak) healthy food purchases
Signposting + menu changes							
Nothwehr 2013 45	Repeat cross‐sectional	Non‐chain owner‐operated full menu, sit‐down restaurants with typical Midwestern fare, Iowa, USA	Enable choice	Environmental restructuring; education	Communication/marketing; environmental/social planning	8	↓ (weak) healthy food purchases
Lee‐Kwan 2013 59	Controlled clinical trial	Non‐franchised small local food establishments that sell ready‐to‐eat food and beverages for off‐premise consumption, Baltimore, USA	Enable choice	Environmental restructuring; education; incentives	Communication/marketing; environmental/social planning	8	↑ (moderate) healthy food purchases
Signposting + health promotion/social marketing campaign							
Fitzgerald 2004 42	Repeat cross‐sectional	Community restaurants varied from counter service to table service, USA	Enable choice	Environmental restructuring; education; persuasion	Communication/marketing; environmental/social planning	6	↓ (weak) sales of ‘heart healthy’ menu items
Acharya 2006 31	Repeat cross‐sectional with control	Restaurant chains (fine dining and moderately priced, family‐style restaurants) (Mexican, upscale pizza, and 40s‐style diner), CA, USA	Enable choice	Environmental restructuring; education; incentives; persuasion	Communication/marketing; environmental/social planning	6	↑ (moderate) healthy food purchases
Horgen and Brownell 2002 58 (*health promotion condition*)	Controlled clinical trial	Delicatessen‐style restaurant (cafeteria), USA	Enable choice	Environmental restructuring; education; persuasion; enablement	Communication/marketing; environmental/social planning	6	↑ (weak) healthy food purchase
Calorie labelling law (*n* = 10)							
Calorie labelling law only							
Bollinger 2011 33	Repeat cross‐sectional with control plus subgroup cohort	Starbucks Cafes, New York City, USA	Provide information	Environmental restructuring; education	Communication/marketing; environmental/social planning; legislation	5	↑ (strong)
Chen 2015 39	Repeat cross‐sectional	Regulated chain or fast food restaurants in King County, USA	Provide information	Environmental restructuring; education	Communication/marketing; environmental/social planning; legislation	5	↑ (weak) saw and used calorie information
Dumanovsky 2011 40, ‡	Repeat cross‐sectional	11 fast‐food chains, New York City, USA	Provide information	Environmental restructuring; education	Communication/marketing; environmental/social planning; legislation	5	↓ (moderate)
Krieger 2013 43, §	Repeat cross‐sectional, retrospective	Restaurants from 10 chains: Subway, McDonald's, Taco del Mar, Taco Time, Starbuck's, Quizno's, Tully's; Jack in the Box, Burger King, Taco Bell, King County, USA	Provide information	Environmental restructuring; education	Communication/marketing; environmental/social planning; legislation	4	↓ (moderate)
Namba 2013 37	Repeat cross‐sectional with control	Large chain fast food restaurants, USA	Provide information	Environmental restructuring; education	Communication/marketing; environmental/social planning; legislation	3	↔ (strong) adults and children
Elbel 2009 34	Repeat cross‐sectional with control	Chain restaurants with >15 establishments – McDonald's, Burger King, Wendy's, KFC in New York City, USA	Provide information	Environmental restructuring; education	Communication/marketing; environmental/social planning; legislation	4	↔ (moderate) adults and children
Elbel 2013 35	Repeat cross‐sectional (before and after legislation) with control cohort (difference in difference design)	Fast food restaurants (McDonald's and Burger King) in Philadelphia (which implemented calorie labelling policies) and Baltimore (which did not and acted as a matched comparison city), USA	Provide information	Environmental restructuring; education	Communication/marketing; environmental/social planning; legislation	5	↔ (moderate)
Finkelstein 2011 36	Repeat cross‐sectional with control	Mexican fast food restaurant chain – Taco Time Northwest, King County, USA	Provide information	Environmental restructuring; education	Communication/marketing; environmental/social planning; legislation	3	↔ (moderate)
Tandon 2011 55	Controlled before‐and‐after study (same participants)	Chain restaurants, King County, USA	Provide information	Environmental restructuring; education	Communication/marketing; environmental/social planning; legislation	4	↔ (weak) children
Calorie labelling law + nutritional recommendation information							
Downs 2013 57	Controlled clinical trial	2 McDonalds restaurants in New York City, USA	Provide information	Environmental restructuring; education	Communication/marketing; environmental/social planning	4	↔ (moderate)
Voluntary calorie labelling (*n* = 1)							
Pulos and Leng 2010 47	Repeat cross‐sectional	Full‐service locally owned (non‐chain) restaurants; ‘casual, midrange’, USA	Provide information	Environmental restructuring; education	Communication/marketing; environmental/social planning	6	↑ (weak) energy, fat and sodium levels of foods purchased
Personalized receipts (*n* = 1)							
Bedard and Kuhn 2013 32	Repeat cross‐sectional with control	Burgerville restaurants (fast‐food chain), California, USA	Provide information	Environmental restructuring; education; persuasion	Communication/marketing	4	↔ (weak)

* Implementation score was determined using a checklist for obesity‐related interventions 25 adapted from workplace interventions 26.

† Energy purchased unless otherwise stated; key: effective (↑), equally effective as a comparison group (↔), effectiveness mixed by outcome or gender (↕), or not effective (↓).

‡ Dumanovsky 2011 and Angell 2012 used the same data set.

§ Krieger 2013 used the same data set as Saelens 2012 (food‐outlet‐level outcomes, Table 2b).

**Table 2b:** Summary of included studies with food‐outlet‐level outcomes (*n* = 7)

Study ID	Study design	Food outlet type	Nuffield intervention ladder	Intervention function	Policy category	Implementation score*	Summary impact (↓↑↔↕) (global quality assessment score)
Award schemes (*n* = 2)							
Gase 2015 52	Cohort	Licensed retail restaurants, Los Angeles County, USA	Restrict choice	Restriction; environmental restructuring	Regulation; environmental/social planning	6	↑ (weak) reduced‐sized portions available and ‘healthier’ children's meals
Bagwell 2014 50	Cohort	Small independent catering outlets, London, UK	Restrict choice	Restriction; environmental restructuring; education	Communication/marketing; regulation; environmental/social planning	2	↑ (weak) ‘healthy’ criteria met by businesses (including catering practices, ‘healthy’ options, health promotion)
Signposting (*n* = 1)							
Signposting + health promotion/social marketing campaign							
Hanni 2009 53	Cohort	Taquerias – privately owned, fast‐food‐style Mexican restaurants, USA	Enable choice	Environmental restructuring; education; incentives; persuasion; enablement; training; modelling	Communication/marketing; environmental/social planning; guidelines	9	↑ (weak) promoting ‘healthier’ food items
Telemarketing of healthy food choices (*n* = 2)							
Wiggers 2001 49, †	Repeat cross‐sectional plus subgroup cohort	Licensed hotels, clubs and nightclubs, New South Wales, Australia	Enable choice	Education	Communication/marketing; environmental/social planning; service provision	6	↑ (weak) serving healthier food options
Licata 2002 44, †	Repeat cross‐sectional plus subgroup cohort	Restaurants and cafés, New South Wales, Australia	Enable choice	Education	Communication/marketing; environmental/social planning; service provision	6	↓ (weak) nutrition‐related health promotion practices
Calorie labelling law (*n* = 2)							
Calorie labelling law only							
Bruemmer 2012 51	Cohort	Chain restaurants with >4 establishments (sit‐down and fast food). Burgers (e.g. McDonalds, Burger King), pizza (e.g. Pizza Hut, Dominos), sandwich/sub (e.g. Subway, Blimpie) or Tex‐Mex (e.g., Taco Time, Taco del Mar), King County, USA	Provide information	Environmental restructuring; education	Communication/marketing; environmental/social planning; legislation	3	↑ (weak) energy content of main meals
Saelens 2012 56, ‡	Controlled before‐and‐after study (retrospective)	Fast food chain restaurants, King County, USA	Provide information	Environmental restructuring; education	Communication/marketing; environmental/social planning; legislation	4	↔ (strong) ‘healthfulness’ of adult and children's menus

* Implementation score was determined using a checklist for obesity‐related interventions 25 adapted from workplace interventions 26. Key: effective (↑); equally effective as the comparison group (↔); effectiveness mixed by outcome or gender (↕); or not effective (↓)

† Licata 2002 and Wiggers 2001 used the same data pool split by different settings.

‡ Saelens 2012 used the same data set as Krieger 2013 (customer‐level outcomes, Table 2a)

### Studies with customer‐level outcomes

#### Trans fat law (*n* = 1)

Only one study (moderate quality, repeat cross‐sectional) investigated the effects of the trans fat law introduced in New York City. Trans fat law was associated with a significant reduction in trans fat content per purchase along with a small, but significant, increase in saturated fat content per purchase. Results did not differ according to the poverty rate of the neighbourhood in which the food outlet was located. However, the effect of the law was inconsistent and varied between fast food chain types.

#### Changing pre‐packed children's meal content (*n* = 1)

One repeat cross‐sectional study (weak quality) investigated the effects of changing the side items included (decrease in portion size of fries and addition of apple slices) in pre‐packed children's meals on energy purchased from these meals 48. The intervention also included a slight change to in‐restaurant and television promotions to include non‐fat chocolate milk in addition to 1% fat plain milk. The study found a decrease in total energy purchased, which was mainly explained by the reduction in energy due to the change in side items. Sales of non‐fat chocolate milk also increased, and sales of regular carbonated drinks decreased from baseline to follow‐up, which resulted in a small but significant contribution to the overall decrease in energy. Of note, there was no change in the percentage of customers choosing the lowest‐energy option. Whilst there did not appear to be any compensatory effects in terms of other pre‐packed meal components, compensatory effects in terms of additional foods were not reported.

#### Price increases for unhealthy choices (*n* = 2)

One strong‐quality controlled trial investigated the effects of two interventions that included price increases of unhealthy menu items: (1) price increase alone and (2) price increase with signposting of the unhealthy options 60. The study found no intervention effect when only a price increase was applied, but when combined with signposting, there was a decrease in unhealthy main dishes ordered 60.

#### Incentives (contingent rewards) (*n* = 1)

A moderate‐quality, brief, cohort study investigated the effects of offering a non‐food incentive (entry to a $10, $50 or $100 lottery) with a smaller portion size option 54. Customers who had intended to order a full‐sized sandwich were offered a half‐sized sandwich plus lottery option (at the same price of the full‐sized sandwich). The proportion of customers who changed their menu choice from a full‐sized to half‐sized sandwich varied by the size of the lottery prize from 5% ($10 lottery) to 8% ($50 lottery) to 22% ($100 lottery) 54.

#### Price reductions for healthier choices (*n* = 2)

One weak‐quality controlled study investigated the effects of two price reduction interventions to promote purchases of healthier options: (1) price reduction alone and (2) price reduction alongside health promotion techniques to highlight the healthier options to customers. Both interventions resulted in a proportional increase in sales of healthier items compared to other items 58.

#### Signposting (*n* = 8)

Eight studies investigated the effects of nine interventions that involved signposting. In three studies, signposting was implemented alone 41, 46, 60; in two studies, signposting was incorporated with menu changes 45, 59, and three studies were of health promotion or social marketing campaigns that included signposting 31, 42, 58.

One controlled trial (strong quality) found that, overall, adding a symbol to menus that identified ‘unhealthy’ main dishes resulted in a decrease in the number of unhealthy main dishes ordered 60. However, when gender effects were explored, it was found that this effect was driven predominately by women.

A repeat cross‐sectional study (weak quality) showed that sales of some healthier items increased after the addition of ‘healthy’ signposting, but for some, sales decreased or were not affected, resulting in no significant overall change in sales of all ‘healthy’ items 41. However, study authors report that the items that showed decreased sales may have been prone to seasonal effects. Another repeat cross‐sectional study (weak quality) found no effect of healthy signposting on the purchase of healthy main meals when added to an existing award intervention 46. This intervention was also culturally tailored; Latino community members helped to translate the messages on small menu stickers into Spanish and provided specific examples of culturally used saturated fats and other ingredients to tailor the national dietary guidelines.

Two studies investigated effects of signposting plus menu changes. One controlled trial (strong quality) found that an intervention promoting new healthier choices was effective in increasing sales of healthy food items 59. However, a repeat cross‐sectional study (weak quality) found that an intervention of table signage promoting new alternative healthier options had no effect on the purchase of healthy choices 45. In the first study 59, food outlets were given support with monetary value in the form of initial stock. In addition, both the menu items and intervention materials aimed to be culturally appropriate through formative research with African–American customers and building rapport with the Korean–American and African–American takeaway owners, e.g. by using and learning greetings in Korean.

Four studies investigated the effects of interventions that primarily aimed to increase customer awareness of healthy options in the participating food outlets. As well as simple menu signposting, these interventions used social marketing or health promotion campaigns to achieve this 31, 42, 53, 58. The intervention investigated by Acharya and colleagues using a repeat cross‐sectional design with control groups (moderate quality) found a significant, small effect on the purchase of healthy menu items compared with controls 31. Holders of campaign discount coupons were 17% more likely to purchase healthy menu items.

A weak‐quality repeat cross‐sectional study investigated an intervention delivered in community food outlets that also included ‘persuasion’ intervention functions (advertisements and articles in local newspaper and newsletters, and promotional material) 42. A trend towards a slight increase in the percentage of healthy items sold was observed, but this did not reach significance. A culturally tailored social marketing campaign, conducted in Mexican–American food outlets, which included the provision of guidelines and training to food outlet owners, incentives (for outlet staff and customers) and newspaper advertising, increased the number of healthier food options provided in the majority of the participating outlets (cohort study; weak quality) 53. In this study, all materials were given to food outlet owners in English and Spanish and were image oriented or comprised simple checklists. Finally, a weak‐quality controlled trial found that displaying in‐store posters listing healthier options led to increases in sales of the healthier options 58.

#### Calorie labelling law (*n* = 10)

The highest number of studies (*n* = 10) assessed the effects of mandatory calorie labelling on menus. Four of these assessed the King County nutrition labelling law 36, 39, 43, 55; four assessed the New York City calorie labelling law 33, 34, 40, 57; one study assessed the Philadelphia calorie labelling law 35; and one study assessed calorie labelling laws across 18 US states and localities 37.

One repeat cross‐sectional study with control (rated strong for quality) showed a statistically significant decrease in average energy purchased following menu calorie labelling in one large coffee chain (Starbucks) compared to control 33. One repeat cross‐sectional study (weak quality) described an increase in the number of customers who reported seeing and acting on the calorie information following introduction of mandatory menu labelling 39. The remaining studies (one weak, five moderate and one strong quality) reported no association between introduction of mandatory menu calorie labelling and average energy purchased 34, 35, 36, 37, 40, 43, 55.

One controlled study (moderate quality) investigated the effects of providing customers with calorie recommendation information before and after the New York City calorie labelling law was implemented 57. The study found that calorie recommendations did not significantly affect food purchases.

#### Voluntary calorie labelling (*n* = 1)

A moderate‐quality repeat cross‐sectional study found that voluntary nutrient (calories, fat, sodium and carbohydrates) labelling in non‐chain food outlets resulted in significant decreases in energy, fat and sodium content of customer purchases, with no change in carbohydrate content 47. The study also found that 71% of customers surveyed reported noticing the nutrition information, with 20% (of all customers) stating that this resulted in choosing a lower‐energy main meal and 17% reported ordering a lower‐fat main meal.

#### Personalized receipts (*n* = 1)

One study (repeat cross‐sectional; weak quality) assessed a receipt‐based intervention 32. The receipts consisted of three components: information, motivation and recommendations. The personalized receipts were associated with an increase in healthier item substitutions that were encouraged by the messages, such as substituting ham for sausage in a breakfast sandwich or substituting frozen yogurt for ice cream. However, there was no significant change in total energy or total fat per transaction. The intervention was also associated with a small increase in revenue (3.2%).

### Studies with food‐outlet‐level outcomes

#### Award schemes (*n* = 2)

Two studies explored the effects of award‐scheme‐type interventions where food outlets received some kind of recognition or certificate for meeting predefined criteria 50, 52. The criteria in each award scheme covered a range of intervention features, and both included restricted choice (e.g. recipe reformulation and default healthy drinks with children's meals). Both studies followed cohort study designs (weak quality) and observed increases in healthier catering practices and healthy options available. However, Bagwell *et al*. 50 found that only a small number of changes were needed for outlets to achieve the award.

#### Signposting (*n* = 1)

One weak‐quality study investigated the effects of a social campaign that included the intervention team working with food outlets to encourage them to add, and signpost, healthier options to their menus 53. The majority of food outlets changed practices by either simply distributing health education materials (94% of 16 food outlets) or introducing or promoting healthier side options (81%), whilst half began promoting healthier main meal options.

#### Telemarketing of healthy food choices (*n* = 2)

Two Australian studies 44, 49 appear to be related to one telemarketing health promotion intervention that included an element of healthy food provision, with one paper focusing on outcomes for hotels, clubs and nightclubs 49 and the other paper on outcomes for restaurants and cafes 44. Both studies used a repeat cross‐sectional study design, with the same cohort of premises evaluated at both time points, and were rated weak for quality. Licata *et al*. 44 found no significant change in the percentage of restaurants and cafes undertaking nutrition‐related health promotion practices between 1997 and 2000, in either the cross‐sectional or cohort samples. However, Wiggers *et al*. 49 found the prevalence of healthy food choices increased significantly in hotels, clubs and nightclubs, in both cross‐sectional and cohort samples.

#### Calorie labelling law (*n* = 2)

Two studies investigated the effects of the King County, USA, calorie labelling law on food‐outlet‐level outcomes. In one cohort study (weak quality), there was a significant decrease in the energy content of main meals available in fast food chain food outlets following the introduction of calorie labelling 51. One strong‐quality controlled study found no association between the introduction of mandatory menu calorie labelling and the ‘healthfulness’ of menus 56.

### Analysis of theoretical framework/behavioural model

Only seven of the 30 studies reported using a theoretical framework or behavioural model, including a consumer behaviour model based on the theory of reasoned action 31, an asset‐based community development approach where community members are active agents of change 53, participatory research 46 and creation of ‘supportive environments’ 49. One study 58 reported using the Health Belief Model, and a matching model 62, which predicts that, because the interval between food choice and eating is short, the proximal satisfaction of a tasty meal would prevail over the distal goal of good health 63. Two studies 45, 59 reported using social cognitive theory; one of these studies also reported using a social marketing approach using the four Ps: product, price, place and promotion 59. Our review protocol 14 included plans to code the use of behaviour change techniques in included interventions, but this endeavour was abandoned *post hoc* because the necessary detail to allow us to do this was only available for seven interventions 31, 45, 46, 49, 53, 58, 59. Attempts were made to contact authors for further information, but only six authors responded to the requests (Table S1). This conclusion was arrived at by experts (V. A. S. and C. A.) with considerable expertise in developing and coding behaviour change techniques in systematic reviews.

**Figure 2:**
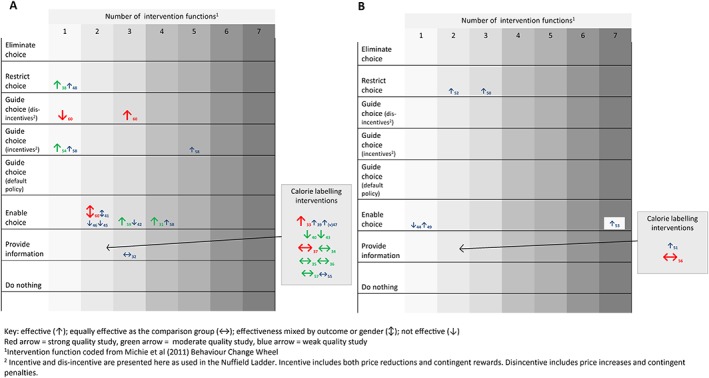
Intervention impact summary by Nuffield intervention ladder category and number of intervention functions for customer‐level outcomes (A) and outlet level outcomes (B)

Figure 2 illustrates the findings from each intervention in the context of the intervention coding according to the Nuffield intervention ladder 18 and the number of intervention functions involved as coded from the Behaviour Change Wheel 29. There is a cluster of interventions lower down the intervention ladder, particularly around providing information, and this mainly includes the calorie labelling law interventions. Evidence for these interventions from the lower end of the Nuffield ladder is mixed. Evidence from the small number of studies higher up the intervention ladder suggests more consistent evidence of effectiveness. The only exception is seen when choices are guided through using price increases, where positive effects were only observed when in conjunction with other intervention elements (which sit further down the ladder). Overall, however, the number of intervention functions does not appear to influence intervention effectiveness.

### Cost‐effectiveness of interventions

There was no cost‐effectiveness evidence reported in any of the included studies.

### Impact of intervention by PROGRESS

Eight studies reported on differential effects of the intervention by population demographics on purchasing behaviour, six of which focused on the impact of calorie labelling. One high‐quality study of mandatory calorie labelling in Starbucks restaurants showed a larger decrease in energy per transaction in ‘zip’ codes with higher‐income and more‐educated residents 33. This was also the only study of mandatory calorie labelling that showed a statistically significant decrease in terms of energy purchased post labelling (approximately 15 cal per purchase). One study found a differential effect of calorie labelling by gender: women but not men significantly reduced mean energy purchased in coffee chains post labelling 43. Some evidence suggests that *awareness* of calorie labelling is highest amongst women and Caucasian, higher‐SES (income and education) and older adults 39, 40.

Two other studies also found differential effects by gender. In a study using a lottery incentive to encourage customers to choose a smaller portion size, women were less likely to take up the offer. There were no effects by age, body mass index or hunger level 54. In another study, women appeared to respond strongly to signposting, whereas for men decreases in unhealthy items purchased were only found when a price increase was added to the signposting 60.

Overall, the limited evidence suggests there are no consistent differential effects (for better or worse) of mandatory calorie labelling in terms of food purchases by gender, age, race and SES. No studies reported data on differential effects of the intervention by occupation, culture/faith/religion or social capital.

## Discussion

### Summary of main findings

Thirty studies describing 34 interventions and meeting the inclusion criteria were identified. Most of these studies (*n* = 27) only collected customer‐level outcome information. Indeed, the evidence is mainly from studies that collected data on meals purchased by adults buying food in specific fast food chains within the USA, which limits the generalizability of the results. Information on the impact of interventions at a food outlet level was scarce and weak in quality. We did not find any information on the impact of interventions on food consumption, by either meal or total daily food intake. The quality of evidence was generally poor, with few high‐quality designs, which limits the strength of the results. Overall, the impact of interventions appears negligible and inconsistent. However, when the impact of interventions was assessed by the level of their intrusiveness, 2As defined by the Nuffield ladder 18 starting with the most intrusive: eliminate choice, restrict choice, guide choice (disincentives), guide choice (incentives), guide choice (default policy), enable choice, provide information and do nothing. patterns emerged. The findings from this review provide useful insight from the best available evidence, which will help to inform future policy and intervention efforts.

Four interventions focused on restricting choice, and all had a positive impact on customer‐level (*n* = 2) and food‐outlet‐level (*n* = 2) outcomes. These types of interventions are sometimes termed ‘health by stealth’, and there is good evidence that such interventions are effective and equitable.

Incentivization, as defined in the Nuffield ladder 18, may be a promising approach to encourage the choice of healthier menu items. Two studies that used a price decrease for healthier options found positive effects on the purchase of healthier food items. Three of four interventions that included price decreases in addition to other intervention functions (targeted at customers and/or the food outlet) found positive effects on healthier food purchases. However, it is unclear what proportion of these positive effects can be attributed to the price changes in these studies. Price increases of unhealthy foods alone were ineffective overall but, when combined with signposting, resulted in a decrease in the purchase of unhealthy items. Eyles *et al*. 64 have reviewed the literature around food pricing strategies and whether they encourage healthy eating habits. Based on modelling studies, they found that taxes on carbonated drinks and saturated fat and subsidies on fruits and vegetables would be associated with beneficial dietary change, with the potential for improved health. The WHO have also concluded that there is a potential to influence consumer purchasing in the desired direction through price policies that address affordability and purchasing incentives; taxes on sugar‐sweetened beverages and targeted subsidies on fruit and vegetables emerge as the policy options with the greatest potential to induce positive changes in consumption. Although there is a dearth of evidence around the effect of policy strategies that aim to promote healthier ready‐to‐eat meals, the results for pricing interventions observed in this review fit with the broader literature 65.

Signposting interventions showed mixed findings. Three signposting‐only studies found mixed or no effect. Six signposting‐plus‐other‐intervention components varied in effectiveness according to study quality. Studies assessed as moderate or strong quality tended to show positive intervention effects, whilst the weak‐quality studies tended to show no or mixed effects. Again, it is unclear what proportion of the effect in these studies can be attributed to the signposting‐only component.

Calorie labelling appears to be associated with an increase in awareness (approximately half of the customers notice labels) and an increase in knowledge of the energy content of fast food menu items. The proportion of customers that notice and act on calorie labelling do tend to purchase fewer calories, but this proportion remains low (less than a third), and no information was available on their subsequent purchases or the impact on overall energy intake.

Results suggest that it is the level of intrusiveness of an intervention, rather than the type of policy function, that determines the impact of the intervention. More ‘intrusive’ interventions (e.g. restrict choice and manipulate price) appear more effective than less intrusive interventions that simply include providing information and enabling choice (e.g. calorie labelling law).

### Strengths and weaknesses of the studies included in the review

There was a dearth of high‐quality studies identified that met the inclusion criteria for this systematic review. The fact that most of the included studies were conducted in chain food outlets in the USA, focused on customer‐level outcomes for adults only and were low to moderate in quality means that caution is required in generalizing and interpreting the results. We appreciate that this type of real‐world public health evaluation is complex but would encourage more researchers and funders to support this type of research, and when doing so to conduct evaluations that can provide information on the cost‐effectiveness and the equity impact of interventions. Although we included every type of outcome in this review, most of those reported were not direct measures of dietary intake or health. Some of the studies reported on the energy value count of food items purchased, but this may not necessarily translate into energy consumed (e.g. because of food sharing and waste), and it cannot be assumed that there were no compensatory effects in food intake at other times in the day. Data on food wastage, food sharing or the act of keeping a proportion of the uneaten food for another meal (e.g. in a ‘doggy bag’) were not collected or reported in the studies we included for review; there is evidence that this is common practice, at least in the USA 66.

The difficulties in identifying behaviour change techniques employed in the studies included in this review may reflect two problems. First, descriptions of interventions in published reports are often poor. This means that the research identified is not replicable and offers limited options for evidence synthesis. This is a widely acknowledged problem 67 and has resulted in the development of the Template for Intervention Description and Replication guidelines for the reporting of interventions 68. Second, because current taxonomies of behaviour change techniques have been inspired by individual behaviour change interventions, it is possible that environmental interventions (e.g. changes to information provided in the menus), like the ones included in this review, are not as well reflected in these taxonomies, making coding difficult.

### Strengths and weaknesses of the review

The primary strength of this systematic review is its scope, in that it assessed the international literature for evidence on this topic, without substantial restriction to any particular intervention, study design or outcome. This novel approach allowed us to comprehensively draw together the best available evidence relating to interventions that promote healthier ready‐to‐eat meals sold by specific food outlets open to the general public. This evidence base can contribute to local and national public health policies given the increasing consumption popularity of ready‐to‐eat meals and international cuisines in many countries 7, 69. That said, this resulted in the assembly of a heterogeneous group of interventions that have a number of different targets for change; some intended to change food outlet practices, and others aimed to change customer behaviour. Previous reviews have focused on calorie labelling 19, 20, 70 or community‐based interventions only 21. Our findings regarding the impact of calorie labelling on sales are in line with these recent systematic reviews 19, 20, 70 that found inconsistent and negligible changes in ‘real‐world’ food outlet settings. Two of these reviews 19, 20 included experimental‐type studies conducted in laboratory and training restaurants, which we did not include (because they were not open to the general public). Calorie labelling in these experimental (efficacy) studies was found to be efficacious. It would appear that these effects are not translated to ‘real‐world’ settings (effectiveness).

### Meaning of the study: possible mechanisms and implications for practitioners and policymakers

We found a preponderance for interventions lower down the Nuffield ladder – particularly in the provide information and enable choice ‘rungs’. This reflects the suggestion made by others that public health policymakers and practitioners may favour those interventions that are less intrusive 71. Unfortunately, our findings, and those of others 71, 72, 73, 74, suggest that these interventions are likely to be less effective and equitable than those higher up the ladder.

The Nuffield ladder was originally developed to help public health practitioners and policymakers determine what level of intervention was ‘proportionate’ for a particular ‘problem’. ‘Intrusiveness’, evidence of effectiveness and the extent of the ‘problem’ addressed are all identified as being important considerations 18. Our findings suggest that interventions higher up the Nuffield ladder are likely to be justified as ones lower down seem of limited effectiveness. We also found some evidence that price‐based and incentive‐based interventions may be particularly promising. However, overall, there is very little evidence on interventions on ‘rungs’ above ‘enable choice’, and further effort is required to both develop and evaluate new approaches.

We also found evidence that less intrusive interventions lower down the Nuffield ladder were more likely to be associated with less equitable effects. The tendency for less intrusive interventions to be less equitable has been discussed by others 71, 75, 76, 77, 78. Whilst this could be interpreted as a limitation, it also serves to highlight that different interventions are required for different population groups and that a range of interventions are required to achieve change across the whole population 71. Although some interventions included in this review included a number of different components, we are not aware of any substantial, multi‐sectorial attempts to achieve wholesale improvement in the healthfulness of the out‐of‐home food sector.

Whole‐system change across the out‐of‐home food sector would require concerted and joined up action across a range of private and public sector organizations. Such action is dependent on political will, which is, in part, dependent on public perceptions of the seriousness of the problem addressed and the effectiveness of the solutions offered 79. Recent changes in the public acceptability of, for example, smoke‐free legislation 80 and taxes on sugar‐sweetened beverages, suggest that public opinion on public health topics is amenable to change.

### Unanswered questions and future research

We found limited evidence of interventions across the full spectrum described in the Nuffield ladder. Further work is required to develop, and evaluate, a wider range of interventions, particularly those higher up the ladder that may be more effective and achieve more equitable effects. This should be conducted in partnership with those working in public health policy and practice.

The quality of evidence included in the review was generally low, limiting the conclusions that can be drawn. Those developing, delivering and evaluating interventions should make greater efforts to ensure that higher‐quality evaluations are conducted, particularly in terms of capturing longitudinal data on outcomes that can be directly related to diet and health. This may require focusing evaluative resources on answering very specific questions well, rather than more diffuse questions less well 81, 82, 83.

We also found that many interventions were very poorly described. Guidance is now available on describing interventions, and intervention components, to facilitate replication and syntheses 68, 84. Researchers and journal editors should make greater efforts to ensure more consistent use of these tools.

Finally, whilst we found some evidence of differential effects of interventions across population sub‐groups, such analyses were mostly absent. Many evaluation studies may have been under‐powered to explore such effects. However, there is good theoretical, and growing empirical, evidence that some interventions – particularly those lower down the Nuffield ladder – are likely to be less effective in those with fewer access to resources 71, 75, 76, 77, 78. Researchers should consider where differential effects may be most likely to occur and design evaluations in such a way that they are able to draw firm conclusions on whether or not such effects occurred.

## Conclusions

Most interventions identified focused on providing information aimed at adults in US fast food chains and collected only customer‐level outcomes; some of these interventions included a function of enabling choice. Overall, most studies were of low or moderate quality. More ‘intrusive’ interventions that restricted or guided choice generally showed a positive impact on food‐outlet‐level and customer‐level outcomes. However, interventions that simply provided information or enabled choice had a negligible impact. Qualitative findings were reported for many studies, particularly around acceptability and process, and these provide useful learning to inform the development of interventions. Interventions involving incentives and more ‘intrusive’ interventions (functions further up the Nuffield ladder, e.g. restrict choice and ‘incentives’) generally showed consistent positive effects on catering practices and the energy value of foods purchased by customers.

## Author contributions

A. A., J. A., V. A. S., A. A. L., H. M., C. S. and M. W. devised the concept for the research. C. S. was responsible for the management of the study. C. S., F. H. B., T. B., H. M. and A. A. L. developed the study protocol and methods, with contributions from A. A., J. A., M. W., V. A. S. and C. A. H. M. and F. H. B. conducted the searches. F. H. B. conducted the screening with assistance from H. M., C. S. and A. A. L. All authors assisted with data extraction. T. B., F. H. B., C. S. and A. R. conducted the data validation, and T. B., F. H. B. and C. S. conducted the data analysis. T. B., F. H. B., C. S., C. A., A. A., J. A., V. A. S. and M. W. contributed to the interpretation of results. C. A. and V. A. S. provided specialized advice (behaviour change theory). F. H. B., T. B. and C. S. drafted the manuscript. All authors have provided critical comments on drafts of the manuscript and have read and approved the final version.

## Funding statement

This study was funded as part of the UK National Institute of Health Research's School for Public Health Research (NIHR SPHR) project: Transforming the ‘foodscape’: development and feasibility testing of interventions to promote healthier takeaway, pub or restaurant food. With additional support from Durham and Newcastle Universities and the NIHR Collaboration for Leadership in Applied Health Research and Care of the South West Peninsula (PenCLAHRC). The School for Public Health Research (SPHR) is funded by the National Institute for Health Research (NIHR). SPHR is a partnership between the Universities of Sheffield, Bristol, Cambridge and Exeter; University College London; The London School for Hygiene and Tropical Medicine; the LiLaC collaboration between the Universities of Liverpool and Lancaster; and Fuse, the Centre for Translational Research in Public Health, a collaboration between Newcastle, Durham, Northumbria, Sunderland and Teesside Universities. Authors F. H. B., C. D. S., H. J. M., W. L. W., A. A., V. A. S. and A. A. L. are members of Fuse; and J. A. and M. W. are funded by the Centre for Diet and Activity Research (CEDAR). Fuse and CEDAR are UK Clinical Research Collaboration (UKCRC) Public Health Research Centres of Excellence. Funding for Fuse and CEDAR comes from the British Heart Foundation, Cancer Research UK, Economic and Social Research Council (ESRC), Medical Research Council, the National Institute for Health Research and the Wellcome Trust, under the auspices of the UKCRC, and is gratefully acknowledged. A. A. is funded by the NIHR as an NIHR Research Professor.

The views expressed are those of the authors and not necessarily those of the aforementioned funders.

## Competing interests

M. W. is funded by NIHR as a Director of its Public Health Research Funding Programme.

## Supporting information

Table S1. Included studies and papers linked to these studies
Table S2. List of excluded studies with reasons
Table S3. Study characteristics
Table S4. Quality assessment of included studies
Table S5. Interventions
Table S6. Results

Supporting info item

Supporting info item

Supporting info item

Supporting info item

Supporting info item

Supporting info item
