# Supplementary material for: The impact of interventions to promote healthier ready‐to‐eat meals (to eat in, to take away or to be delivered) sold by specific food outlets open to the general public: a systematic review
Source: Obes Rev. 2016 Nov 29;18(2):227–46. doi: 10.1111/obr.12479 (PMC5244662; doi:10.1111/obr.12479)
Supplement: Supplementary file 1 — Supporting info item [file OBR-18-227-s001.docx]

**Table S1: Included studies and papers linked to these studies**

*Studies in* ***bold*** *type are the main study papers in cases where there are linked papers*

1. Acharya, R. N., P. M. Patterson, E. P. Hill, T. G. Schmitz and E. Bohm (2006). "An Evaluation of the "TrEAT Yourself Well" Restaurant Nutrition Campaign." Health Education & Behavior 33(3): 309-324.
2. Angell, S. Y., L. K. Cobb, C. J. Curtis, K. J. Konty and L. D. Silver (2012). "Change in Trans Fatty Acid Content of Fast-Food Purchases Associated With New York City's Restaurant Regulation: A Pre-Post Study." Annals of Internal Medicine 157(2): 81-86.
3. Bagwell, S. (2014). "Healthier catering initiatives in London, UK: an effective tool for encouraging healthier consumption behaviour?" Critical Public Health 24(1): 35-46.
4. **Bedard, K. and P. Kuhn (2013). Making Nutritional Information Digestible: Effects of a Receipt-Based Intervention on Restaurant Purchases. National Bureau of Economic Research Working Paper No. 19654. Cambridge, MA, National Bureau of Economic Research.**

Bedard, K. and P. Kuhn (2014). "Micro-marketing healthier choices: Effects of personalized ordering suggestions on restaurant purchases." Journal of Health Economics 39: 106-122.

1. **Bollinger B, Leslie P, Sorensen T. Calorie Posting in Chain Restaurants. *American Economic Journal: Economic Policy* 2011; 3:91-128.**

Bollinger, B., P. Leslie and A. Sorensen (2010). Calorie Posting in Chain Restaurants. National Bureau of Economic Research Working Paper No. w15648. Cambridge, MA, National Bureau of Economic Research.

1. Bruemmer B, Krieger J, Saelens BE, Chan N. Energy, saturated fat, and sodium were lower in entre´es at chain restaurants at 18 months compared with 6 months following the implementation of mandatory menu labeling regulation in King County, Washington. J Acad Nutr Diet 2012;112(8):1169–76.
2. Chen, R., et al. (2015). "Changes in Awareness and Use of Calorie Information After Mandatory Menu Labeling in Restaurants in King County, Washington." American Journal of Public Health 105(3): 546-553.
3. Downs, J. S., J. Wisdom, B. Wansink and G. Loewenstein (2013). "Supplementing menu labeling with calorie recommendations to test for facilitation effects." American journal of public health 103(9): 1604-1609.
4. Dumanovsky, T., C. Y. Huang, C. A. Nonas, T. D. Matte, M. T. Bassett and L. D. Silver (2011). "Changes in energy content of Lunchtime purchases from fast food restaurants after introduction of calorie labelling: cross sectional customer surveys." BMJ (British Medical Journal) 343(7818): 299.
5. **Elbel, B., R. Kersh, V. L. Brescoll and L. B. Dixon (2009). "Calorie labeling and food choices: a first look at the effects on low-income people in New York City." Health Affairs (Project Hope) 28(6): 1110-1121.**

Elbel, B. (2011). "Consumer estimation of recommended and actual calories at fast food restaurants." Obesity 19(10): 1971-1978.

Elbel, B., J. Gyamfi and R. Kersh (2011). "Child and adolescent fast-food choice and the influence of calorie labeling: A natural experiment." International Journal of Obesity 35(4): 493-500.

Vadiveloo, M., L. B. Dixon and B. Elbel (2011). "Consumer responses to menu labeling legislation in New York City - Have purchasing patterns been affected?" FASEB Journal 25.

Vadiveloo, M. K., L. B. Dixon and B. Elbel (2011). "Consumer purchasing patterns in response to calorie labeling legislation in New York City." The international journal of behavioral nutrition and physical activity 8: 51.

1. **Elbel, B., et al. (2013). "Calorie labeling, fast food purchasing and restaurant visits." Obesity 21(11): 2172-2179.**

Taksler, G. B. and B. Elbel (2014). "Calorie labeling and consumer estimation of calories purchased." The international journal of behavioral nutrition and physical activity 11: 91.

1. Eldridge, A. L., M. P. Snyder, N. G. Green Faus and K. Kotz (1997). "Development and Evaluation of a Labeling Program for Low-Fat Foods in a Discount Department Store Foodservice Area." Journal of Nutrition Education 29(3): 159-161.
2. Finkelstein, E. A., K. L. Strombotne, N. L. Chan and J. Krieger (2011). "Mandatory Menu Labeling in One Fast-Food Chain in King County, Washington." American Journal of Preventive Medicine 40(2): 122-127.
3. Fitzgerald, C. M., S. Kannan, S. Sheldon and K. A. Eagle (2004). "Effect of a promotional campaign on heart-healthy menu choices in community restaurants.[Erratum appears in J Am Diet Assoc. 2004 Jun;104(6):1013]." Journal of the American Dietetic Association 104(3): 429-432.
4. Gase, L. N., et al. (2015). "What menu changes do restaurants make after joining a voluntary restaurant recognition program?" Appetite 89: 131-135.
5. Hanni, K. D., E. Garcia, C. Ellemberg and M. Winkleby (2009). "Steps to a Healthier Salinas: Targeting the Taqueria: Implementing Healthy Food Options at Mexican American Restaurants." Health Promotion Practice 10(2 suppl): 91S-99S.
6. Horgen, K. B. and K. D. Brownell (2002). "Comparison of price change and health message interventions in promoting healthy food choices." Health Psychology 21(5): 505-512.
7. Krieger, J. W., N. L. Chan, B. E. Saelens, M. L. Ta, D. Solet and D. W. Fleming (2013). "Menu Labeling Regulations and Calories Purchased at Chain Restaurants." American Journal of Preventive Medicine 44(6): 595-604.
8. **Lee-Kwan, S. H., S. Goedkoop, R. Yong, B. Batorsky, V. Hoffman, J. Jeffries, M. Hamouda and J. Gittelsohn (2013). "Development and implementation of the Baltimore healthy carry-outs feasibility trial: process evaluation results." BMC public health 13: 638.**

Lee, S. H. (2012). Changing the food environment in baltimore city: Impact of an intervention to improve carry-outs in Low-income neighborhoods, Johns Hopkins University.

Lee, S. H., H. Kim, R. Yong, M. Hamouda, J. Shon, J. H. Park and J. Gittelsohn (2012). "Environmental intervention in carryouts increases sales of healthy menu items in low-income urban setting." FASEB Journal 26; (32):7.

Lee-Kwan, S. H., R. Yong, S. N. Bleich and J. Gittelsohn (2013). "Increased healthy food purchasing associated with exposure to carry-out intervention in low-income urban setting." FASEB Journal 27.

1. Licata, M., K. Gillham and E. Campbell (2002). "Health promotion practices of restaurants and cafes in Australia: changes from 1997 to 2000 using an annual telemarketing intervention." Health Promotion International 17(3): 255-262.
2. Wiggers, J., R. Considine, T. Hazell, M. Haile, M. Rees and J. Daly (2001). "Increasing the Practice of Health Promotion Initiatives by Licensed Premises." Health Education & Behavior 28(3): 331-340.
3. Namba, A., A. Auchincloss, B. L. Leonberg and M. G. Wootan (2013). "Exploratory analysis of fast-food chain restaurant menus before and after implementation of local calorie-labeling policies, 2005-2011." Preventing chronic disease 10: E101.
4. Nothwehr, F. K., L. Snetselaar, J. Dawson and U. Schultz (2013). "Promoting Healthy Choices in Non-Chain Restaurants: Effects of a Simple Cue to Customers." Health Promotion Practice 14(1): 132-138.
5. Pandya (2013). Examining the effects of a healthy restaurant intervention on customers’ purchases of healthier food options in Latino family-owned restaurants, University of Kansas.
6. Pulos, E. and K. Leng (2010). "Evaluation of a voluntary menu-labeling program in full-service restaurants." American Journal of Public Health 100(6): 1035-1039.
7. Reimann, M., et al. (2015). "Leveraging the happy meal effect: Substituting food with modest nonfood incentives decreases portion size choice." Journal of Experimental Psychology: Applied 21(3): 276-286.
8. Saelens, B. E., N. L. Chan, J. Krieger, Y. Nelson, M. Boles, T. A. Colburn, K. Glanz, M. L. Ta and B. Bruemmer (2012). "Nutrition-Labeling Regulation Impacts on Restaurant Environments." American Journal of Preventive Medicine 43(5): 505-511.
9. Shah, A. M., et al. (2014). "Surcharges plus unhealthy labels reduce demand for unhealthy menu items." Journal of Marketing Research 51(6): 773-789.
10. Tandon, P. S., C. Zhou, N. L. Chan, P. Lozano, S. C. Couch, K. Glanz, J. Krieger and B. E. Saelens (2011). "The Impact of Menu Labeling on Fast-Food Purchases for Children and Parents." American Journal of Preventive Medicine 41(4): 434-438.
11. Wansink, B. and A. S. Hanks (2014). "Calorie reductions and within-meal calorie compensation in children's meal combos." Obesity 22(3): 630-632.
