# Supplementary material for: The impact of interventions to promote healthier ready‐to‐eat meals (to eat in, to take away or to be delivered) sold by specific food outlets open to the general public: a systematic review
Source: Obes Rev. 2016 Nov 29;18(2):227–46. doi: 10.1111/obr.12479 (PMC5244662; doi:10.1111/obr.12479)
Supplement: Supplementary file 2 — Supporting info item [file OBR-18-227-s002.docx]

## Table S2: List of excluded studies with reasons

| **Reference** | **Reason for exclusion** |
| --- | --- |
| Aase, S. (2009). "Taking trans fat off the menu: what you can learn from trans-fat bans at Sheikh Khalifa Medical City and the Cleveland Clinic." *Journal of the American Dietetic Association* 109(7): 1148-1149, 1951. | Not primary research |
| Abel, M. L., et al. (2015). "Consumer Understanding of Calorie Labeling: A Healthy Monday E-Mail and Text Message Intervention." Health Promotion Practice 16(2): 236-243. | Setting not in included OHFO |
| Allan, J. L., et al. (2015). "Snack purchasing is healthier when the cognitive demands of choice are reduced: A randomized controlled trial." Health Psychology 34(7): 750-755. | Setting not in included OHFO |
| Allen, K. N., et al. (2007). "Effectiveness of nutrition education on fast food choices in adolescents." *The Journal of school nursing: the official publication of the National Association of School Nurses* 23(6): 337-341. | Setting not in included OHFO |
| Allison, J. T. (2005). "ARAMARK takes Baylor's retail food program to new heights. ARAMARK Healthcare Management Services raises customer satisfaction and revenue levels for Baylor Health Care System through management of its retail food program." *Healthcare Executive* 20(3): 32-33. | No outcomes reported or not outcome of interest |
| Anon. (2000). "Quick take: "tear out" sheets for quick reference. 20th century served up safer, healthier food." *Consultant* (00107069) 40(12): 2069-2069. | Unable to find full text |
| Anon. (2005). "Fast food really can be unhealthy...and new dietary guidelines." *Child Health Alert* 23: 3-4. | Unable to find full text |
| Anon. (2012). "Heart-healthy menu choices now clear. Logo makes it easy to spot low-fat, low-calorie entrees." *Harvard Heart Letter: from* *Harvard Medical School* 23(2): 7. | Unable to find full text |
| Anon. (2013). "Fast-Food Consumption Dips." *Tufts University Health & Nutrition Letter* 31(3): 2-2. | No OHFO intervention e.g. survey data |
| Antoniolli, R. E., et al. (2014). "Total lunchtime fast food purchases were lower in sodium and saturated fat when nutritionally promoted fast foods were ordered instead of traditional fast foods: A pilot study." Nutrition & Dietetics 71(1): 41-45. | No before/after measures |
| Auchincloss, A. H., et al. (2013). "Barriers and facilitators of consumer use of nutrition labels at full-service [corrected] restaurant chains." Public Health Nutrition 16(12): 2138-2145. | No before/after measures |
| Auchincloss, A. H., et al. (2013). "Customer Responses to Mandatory Menu Labeling at Full-Service Restaurants." American Journal of Preventive Medicine 45(6): 710-719. | No before/after measures |
| Auchincloss, A. H., et al. (2014). "Nutritional Value of Meals at Full-service Restaurant Chains." Journal of Nutrition Education & Behavior 46(1): 75-81. | No before/after measures |
| Backman, D., et al. (2011). "Catering trucks in California promote healthful eating in low-wage worksites." *Journal of Nutrition Education and Behavior* 43(4 Suppl 2): S155-157. | Setting not in included OHFO |
| Barrett, J. (2003). "Fast food need not be fat food." Newsweek 142(15): 73-74. a. http://www.newsweek.com/health-fast-food-need-not-be-fat-food-138517 | No outcomes reported or not outcome of interest |
| Bauer, K. W., et al. (2009). "Socio-environmental, personal and behavioural predictors of fast-food intake among adolescents." *Public Health Nutrition* 12(10): 1767-1774. | No OHFO intervention e.g. survey data |
| Beaulieu, D. and G. Godin (2012). "Staying in school for lunch instead of eating in fast-food restaurants: results of a quasi-experimental study among high-school students." *Public Health Nutrition* 15(12): 2310-2319. | Setting not in included OHFO |
| Becker, N. (2010). "Grassroots Efforts behind Restaurant Menu Labeling Legislation." *Journal of the American Dietetic Association* 110(11): 1633-1633. | Not primary research |
| Bell, C., et al. (2013). "Healthier choices in an Australian health service: a pre-post audit of an intervention to improve the nutritional value of foods and drinks in vending machines and food outlets." BMC health services research 13: 492. | Setting not in included OHFO |
| Berman, M. and R. Lavizzo-Mourey (2008). "Obesity prevention in the information age: caloric information at the point of purchase." *Journal of the American Medical Association* 300(4): 433-435. | Not primary research |
| Biediger-Friedman, L., et al. (2013). "Different settings, common solution: A healthy menu designation program in restaurants and institutions." *FASEB Journal* 27. | No before/after measures |
| Biediger-Friedman, L., et al. (2013). "Different settings, common solution: A healthy menu designation program in restaurants and institutions." FASEB Journal 27. | No before/after measures |
| Black, E. A. (2014). "Menu labeling: the unintended consequences to the consumer." Food and drug law journal 69(4): 531-554. | Not primary research |
| Blair, A. M., et al. (2011). "Restaurant Challenge Offers Healthful Meal Options and Builds Diabetes Awareness." *Diabetes Educator* 37(4): 581-588. | No before/after measures |
| Bleich, S. N., et al. (2015). "Calorie changes in chain restaurant menu items: implications for obesity and evaluations of menu labeling." American Journal of Preventive Medicine 48(1): 70-75. | No before/after measures |
| Block, J. P. and C. A. Roberto (2014). "Calorie labeling on menus and menu boards--reply." Jama 312(24): 2689. | Not primary research |
| Block, J. P. and C. A. Roberto (2014). "Potential benefits of calorie labeling in restaurants." JAMA - Journal of the American Medical Association 312(9): 887-888. | Not primary research |
| Blumenthal, K. and K. G. Volpp (2010). "Enhancing the effectiveness of food labeling in restaurants." *Journal of the American Medical Association* 303(6): 553-554. | Not primary research |
| Bove, A., et al. (2006). "Burger king and subway: Key nutrients, glycemic index, and glycemic load of nutritionally promoted items." *Diabetes Educator* 32(5): 675-690. | No OHFO intervention e.g. survey data |
| Bowers, K. M. and S. Suzuki (2014). "Menu-labeling usage and its association with diet and exercise: 2011 BRFSS Sugar Sweetened Beverage and Menu Labeling module." Preventing chronic disease 11: 130231. | No before/after measures |
| Boyle, M. (2004). "Can you really make fast food healthy?" *Fortune* 150(3): 134-138, 140.  a.http://money.cnn.com/magazines/fortune/fortune_archive/2004/08/09/377886/ | Not primary research |
| Breck, A., et al. (2014). "Who reports noticing and using calorie information posted on fast food restaurant menus?" Appetite 81: 30-36. | Not primary research |
| Brissette, I., et al. (2013). "Predictors of Total Calories Purchased at Fast-food Restaurants: Restaurant Characteristics, Calorie Awareness, and Use of Calorie Information." *Journal of Nutrition Education & Behavior* 45(5): 404-411. | No before/after measures |
| Britt, J. W., et al. (2011). "Feasibility of Voluntary Menu Labeling among Locally Owned Restaurants." *Health Promotion Practice* 12(1): 18-24. | No before/after measures |
| Brochu, P. M. and J. F. Dovidio (2014). "Would You Like Fries (380 Calories) With That? Menu Labeling Mitigates the Impact of Weight-Based Stereotype Threat on Food Choice." Social Psychological & Personality Science 5(4): 414-421. | Setting not in included OHFO |
| Anon. (2011). "Fast food info becoming more comprehensive." *Vegetarian Journal* 30(1): 5-5. a. http://www.vrg.org/journal/vj2011issue1/2011_issue1_letters.php | Not primary research |
| Caraher, M., et al. (2013). "The planning system and fast food outlets in London: Lessons for health promotion practice." *Revista Portuguesa de Saude Publica* 31(1): 49-57.  a. http://www.sciencedirect.com/science/article/pii/S0870902513000060 | Not English language |
| Carter, C. (2011). "Restaurants that get it right." *Men's Health* (10544836) 26(9): 108-108. a. http://www.menshealth.com/nutrition/restaurants-get-it-right | Not primary research |
| Caterson, I., et al. (2014). "8700-menu labelling regulation in New South Wales Australia-social marketing campaign and results." Obesity Reviews 15: 269. | Abstract only |
| Chan, N. L., B. Bruemmer, D. Solet, B. E. Saelens and J. Krieger (2009). Impact of nutrition labels on customers of fast food and coffee chains in Seattle/King County. *137th annual meeting of the American Public Health Association*. Philadelphia, PA. | Unable to find full text |
| Chan, N., J. Krieger, B. Saelens, M. Ta and D. Solet (2012). Over one year later, menu labeling policy associated with drop in high calorie purchases at fast food chains in Seattle, King County, Washington*. Robert Wood Johnson Foundation Healthy Eating Research Conference*, Austin, TX. | Unable to find full text |
| Chand, A., et al. (2012). "Availability and accessibility of healthier options and nutrition information at New Zealand fast food restaurants." *Appetite* 58(1): 227-233. | No before/after measures |
| Chaufan, C., et al. (2011). "Food for thought: Menu labeling as obesity prevention public health policy." *Critical Public Health* 21(3): 353-358. | Not primary research |
| Chen, R., et al. (2011). "Partnering with REACH to create a "diabetes-friendly" restaurant: a restaurant owner's experience." *Progress in Community Health Partnerships: Research, Education, and Action* 5(3): 307-312. | No before/after measures |
| Chiang, P. H., et al. (2011). "Fast-food outlets and walkability in school neighbourhoods predict fatness in boys and height in girls: a Taiwanese population study." *Public Health Nutrition* 14(9): 1601-1609. | No OHFO intervention e.g. survey data |
| Cicero, K. (2011). "Take the kids out to eat!" *Parents* (10836373) 86(4): 66-72. a. http://www.parents.com/recipes/nutrition/best-family-restaurants-2011/ | Not primary research |
| Cohn, E. G., et al. (2012). "Calorie postings in chain restaurants in a low-income urban neighborhood: measuring practical utility and policy compliance." *Journal of Urban Health* 89(4): 587-597. | No before/after measures |
| Collier, R. (2013). "Nutrition information noticed in restaurants if on menu." *Canadian Medical Association Journal* 185(11): 945-945. a. http://www.cmaj.ca/content/185/11/945.extract | Not primary research |
| Conis, E. (2011). "Company promises healthful fast food." *Los Angeles Times -Southern California Edition* (Front Page): E1. | Unable to find full text |
| Conkin, C., et al. (2012). "Adolescent Fast Food Choices in the Era of the Menu Boards with Nutritional Information: A Study to Maximize Effectiveness." *Journal of the Academy of Nutrition & Dietetics* 112: A86-A86. | Setting not in included OHFO |
| Cunningham, E. (2003). "Where can I find resources on food labeling in a restaurant setting?" *Journal of the American Dietetic Association* 103(2): 199. | Not primary research |
| Davies, E. (2012). "Two US cities are to display calorie counts on vending machines." *British Medical Journal* 345(e6884). | No outcomes reported or not outcome of interest |
| Diller, P. A. and S. Graff (2011). "Regulating Food Retail for Obesity Prevention: How Far Can Cities Go?" *Journal of Law, Medicine & Ethics* 39: 89-93. | Not primary research |
| Downs, J. S. (2013). "Does "healthy" fast food exist? the gap between perceptions and behavior." Journal of Adolescent Health 53(4): 429-430. | Not primary research |
| Dubost, J. (2011). "Restaurant foods and stated energy contents.” *Journal of the American Medical Association* 306(15):1655-1656 | Not primary research |
| Dumanovsky, T., et al. (2010). "Consumer awareness of fast-food calorie information in New York City after implementation of a menu labeling regulation." *American Journal of Public Health* 100(12): 2520-2525. | No outcomes reported or not outcome of interest |
| Economos, C. D., et al. (2009). "A community-based restaurant initiative to increase availability of healthy menu options in Somerville, Massachusetts: Shape Up Somerville." *Preventing Chronic Disease* 6(3): A102. | No before/after measures |
| Eden, B., et al. (2012). "In reply to article 'Sales of healthy choices at fast food restaurants in Australia' by Wellard, Glasson and Chapman." *Health Promotion Journal of Australia: Official Journal of Australian Association of Health Promotion Professionals* 23(2): 158. | Not primary research |
| Elbel, B., et al. (2015). "New York City healthy happy meals bill: Potential impact on fast food purchases." American Journal of Preventive Medicine 49(4): e45-e46. | No before/after measures |
| Eldridge, S. and D. Woodward (2003). "A fresh mix. Meadow Lakes entices residents with a menu of lighter, healthier fare." *Contemporary Longterm Care* 26(10): 30-31. | Unable to find full text |
| Eskin, S. B. and S. Hermanson (2004). "Nutrition labeling at fast-food and other chain restaurants." *Issue Brief (Public Policy Institute (American Association of Retired Persons)* IB71:1-6. | Unable to find full text |
| Fitzpatrick, M. P., et al. (1997). "Lower-fat menu items in restaurants satisfy customers." *Journal of the American Dietetic Association* 97(5): 510-514. | No before/after measures |
| Fotouhinia, M. (2011). "Evaluation of menu nutrition labeling on clients food choices and calorie consumption in full service restaurants." *Annals of Nutrition and Metabolism* 58: 44. | Setting not in included OHFO |
| Fotouhinia, Y. M., et al. (2013). "Consumer acceptability and effectiveness of six menu calorie labeling formats on food choices using mobile tablet technology." *Annals of Nutrition and Metabolism* 63: 882. | No OHFO intervention e.g. survey data |
| Fox, M. (2015). "New menu labeling requirements: Academy advocacy expands opportunities." Journal of the Academy of Nutrition and Dietetics 115(5): 707-708. | Not primary research |
| French, S., R. Jeffery, M. Story, K. Breitlow, J. Baxter, P. Hannan and M. Snyder (2001). "Pricing and promotion effects on low-fat vending snack purchases: the CHIPS Study." *American Journal of Public Health* 91(1): 112-117. | Setting not in included OHFO |
| French, S., R. Jeffery, M. Story, P. Hannan and M. Snyder (1997). "A pricing strategy to promote low-fat snack choices through vending machines." *American Journal of Public Health* 87(5): 849-851. | Setting not in included OHFO |
| Gallick, B. and L. Lee (2009). ""Cheesy Pizza": The Pizza Project." *Early Childhood Research & Practice* 11(2).  a. http://ecrp.uiuc.edu/v11n2/gallick.html | Setting not in included OHFO |
| Geiger, S. M. (2012). "Finding Fit Fare at Restaurants." *Environmental Nutrition* 35(4): 1-6. | Unable to find full text |
| Gordon, C. and R. Hayes (2012). "Counting Calories: Resident Perspectives on Calorie Labeling in New York City." *Journal of Nutrition Education & Behavior* 44(5): 454-458. | No before/after measures |
| Green, K. L., et al. (1993). "Evaluation of the Heart Smart Restaurant Program in Saskatoon and Regina, Saskatchewan." *Canadian Journal of Public Health*. *Revue Canadienne de Sante Publique* 84(6): 399-402. | No before/after measures |
| Haapala, I., et al. (2011). "Improving the quality of meals eaten or prepared outside the home." *Public Health Nutrition* 14(2): 191-192. | Not primary research |
| Hammond, D., et al. (2013). "A randomized trial of calorie labeling on menus." Preventive Medicine 57(6): 860-866. | Setting not in included OHFO |
| Hanni, K., E. Mendoza, J. Snider and M. Winkleby (2007). "A methodology for evaluating organizational change in community-based chronic disease interventions." *Preventing Chronic Disease* 4(4): A105. | No before/after measures |
| Hanratty, B., et al. (2012). "'McDonalds and KFC, it's never going to happen': the challenges of working with food outlets to tackle the obesogenic environment." *Journal of Public Health* 34(4): 548-554. | No OHFO intervention e.g. survey data |
| Harnack, L. J. (2006). "Availability of Nutrition Information on Menus at Major Chain Table-Service Restaurants." *Journal of the American Dietetic Association* 106(7): 1012-1015. | No before/after measures |
| Harnack, L., S. French, J. Oakes, M. Story, R. Jeffery and S. Rydell (2008). "Effects of calorie labeling and value size pricing on fast food meal choices: Results from an experimental trial." *International Journal of Behavioral Nutrition and Physical Activity* 5(1): 1-13. | Setting not in included OHFO |
| Heiman, A. and O. Lowengart (2014). "Calorie information effects on consumers' food choices: Sources of observed gender heterogeneity." Journal of Business Research 67(5): 964-973. | Setting not in included OHFO |
| Heller, T. (1994). "The Heeley Health Project." *Occasional paper (Royal College of General Practitioners)* (64): 11-13. | No OHFO intervention e.g. survey data |
| Herreria, J. (1998). "Healthy Heart endeavors appeal to the surrounding Sheffield, Ala., area." *Profiles in Healthcare Marketing* 14(4): 24-26. | Unable to find full text |
| Holmes, A. S., et al. (2013). "Effect of different children's menu labeling designs on family purchases." Appetite 62: 198-202. | Setting not in included OHFO |
| Hong, K. and H. Joung (2009). "Restaurateur's Willingness to Participate in the Healthy Restaurant Program in Seoul." *Korean Journal of Nutrition* 42(3): 268-277. | Not English language |
| Howlett, E. A., et al. (2009). "Coming to a restaurant near you? Potential consumer responses to nutrition information disclosure on menus." *Journal of Consumer Research* 36(3): 494-503. | Setting not in included OHFO |
| Hurley, J. and B. Liebman (2010). "Sandwich, soup, & salad smarts: a guide to quick-café menus." *Nutrition Action Health Letter* 37(9): 12-15.  a.http://www.thefreelibrary.com/Sandwich,+soup,+%26+salad+smarts%3A+a+guide+to+quick-cafe+menus.-a0241779620 | Not primary research |
| Jebb, S. A. (2011). "Calorie labelling on the high street." *British Medical Journal* 343: d4502. | Not primary research |
| Jeffery, R. W., et al. (1994). "An environmental intervention to increase fruit and salad purchases in a cafeteria." *Preventive Medicine* 23(6): 788-792. | Setting not in included OHFO |
| Jeffries, J. K., et al. (2013). "Preferences for Healthy Carryout Meals in Low-Income Neighborhoods of Baltimore City." *Health Promotion Practice* 14(2): 293-300. | No OHFO intervention e.g. survey data |
| Jones, J. M. (2014). "Free toy promotions, fast food children’s meals and social responsibility: Examining the effects of toy value, nutrition information and moderating variable." Journal of Managerial Issues 26(3): 240-258. | Setting not in included OHFO |
| Joung, H. (2008). Effectiveness evaluation of Healthy Restaurant Program, Seoul National University School of Public Health and Management Centre for Health Promotion. | Not English language |
| Kenney, J. J. (2009). "Which menu labeling law is best?" *Communicating Food for Health* 57-59.  a. http://www.communicatingfoodforhealth.com/wp-content/uploads/minutes-agendas-newsletters/Newsletter_2009-09-01.pdf | Not primary research |
| Kerins, C., et al. (2015). "Adressing the obesity epidemic through a novel user-friendly approach to menu labelling in the west of ireland." Obesity Facts 8: 46. | Abstract only |
| King, L. E. (2013). "Corralling your cravings: Promoting fast food nutrition awareness in butte, MT." *Journal of Investigative Medicine* 61 (1): 164. | Setting not in included OHFO |
| Krieger, J. (2010). Chain restaurant nutrition labeling: An overview of implementation and evaluation in New York City, Portland/Multnomah County, Seattle/King County, and Philadelphia. *138th annual meeting of the American Public Health Association*, Denver, CO. | Unable to find full text |
| Lachat, C., et al. (2011). "Essential actions for caterers to promote healthy eating out among European consumers: results from a participatory stakeholder analysis in the HECTOR project." *Public Health Nutrition* 14(2): 193-202. | No OHFO intervention e.g. survey data |
| Larson, N. and M. Story (2009). Menu labeling: does providing nutrition information at the point of purchase affect consumer behavior? Princeton (NJ), RobertWood Johnson Foundation. | Not primary research |
| Lee, S., M. Rowan, L. Dennisuk, A. Klassen, K. D Frick, L. Powell and J. Gittelsohn (2011). "Pilot environmental intervention trial in carry-outs in low-income neighborhoods of Baltimore City." *FASEB Journal* 25. | No before/after measures |
| Lee-Kwan, S. H., et al. (2014). "Restaurant menu labeling use among adults - 17 States, 2012." MMWR: Morbidity & Mortality Weekly Report 63(27): 581-584. | Not primary research |
| Leontos, C. (2001). "Eating out healthfully." *Diabetes Self-Management* 18(1): 9-12. | Unable to find full text |
| Levin, S. (1996). "Pilot Study of a Cafeteria Program Relying Primarily on Symbols to Promote Healthy Choices." *Journal of Nutrition Education* 28(5): 282-285. | Setting not in included OHFO |
| Lipscomb, R. (2011). "Identifying Opportunities: A Report on the 2010 American Dietetic Association Environmental Scan on Restaurant Menu Labeling." *Journal of the American Dietetic Association* 111(4): 511-516. | No OHFO intervention e.g. survey data |
| Lloyd, B. and S. Dumbrell (2011). "Modifying the food supply at a community swimming pool: a case study." *Health Promotion Journal of Australia* 22(1): 22-26. | No before/after measures |
| Loewenstein, G. (2011). "Confronting reality: pitfalls of calorie posting." *American Journal of Clinical Nutrition* 93(4): 679-680. | Not primary research |
| Long, M. W., et al. (2015). "Systematic review and meta-analysis of the impact of restaurant menu calorie labeling." American Journal of Public Health 105(5): e11-e24. | Not primary research |
| Love, R. (2008). "Access to healthy food in a low-income urban community: a service-learning experience." *Public Health Reports* 123(2): 244-247. | No OHFO intervention e.g. survey data |
| Lucan, S. C. and J. J. DiNicolantonio (2014). "Standardizing portion sizes may not benefit human or environmental health." American Journal of Public Health 104(12): e8. | Not primary research |
| Macaskill, L. A., et al. (2003). "Eat Smart! Ontario's Healthy Restaurant Program: a survey of participating restaurant operators." *Canadian Journal of Dietetic Practice and Research* 64(4): 202-207. | No before/after measures |
| Macaskill, L., et al. (2000). "An evaluability assessment to develop a restaurant health promotion program in Canada." *Health Promotion International* 15(1): 57-69. | No before/after measures |
| McDonald, D. (2012). "Challenging Ronald: McDonald versus McDonald's." *Journal of Paediatrics & Child Health* 48(2): 103-105. | No OHFO intervention e.g. survey data |
| McKevith, B. and J. Buttriss (2002). "Improving provision of nutrition information in catering outlets and for food sold loose." *Nutrition Bulletin* 27(4): 237-239. | No before/after measures |
| Mello, M. M. (2009). "New York City's war on fat." *The New England Journal of Medicine* 360(19): 2015-2020. | Not primary research |
| Mello, M. M. (2012). "Nutrition: Ejecting trans fat from New York City restaurants." *Nature Reviews Endocrinology* 8(11): 633-635. | Not primary research |
| Mergler, B. and S. Jasti (2011). "Use of calorie labeling on menus in New York City fast food restaurants among college students." *FASEB Journal* 25. | No before/after measures |
| Meyer, K. A., et al. (2014). "Sociodemographic differences in fast food price sensitivity." JAMA Internal Medicine 174(3): 434-442. | No OHFO intervention e.g. survey data |
| Middleton, G. and K. Evans (2013). "Exploring the possibilities of transforming Indian cuisine: Findings from the Healthy Takeaways project in Lincolnshire (Part I)." Proceedings of the Nutrition Society 72: E270. | No before/after measures |
| Molloy, M. (2002). "Practice notes: strategies in health education. Winner's Circle Healthy Dining Program." *Health Education and Behavior* 29: 406-408. | Not primary research |
| Murphy, S., et al. (1994). "A formative evaluation of the Welsh Heartbeat Award Scheme." *Nutrition and Health* 9(4): 317-327. | No before/after measures |
| Nago, E. S., et al. (2014). "Association of Out-of-Home Eating with Anthropometric Changes: A Systematic Review of Prospective Studies." Critical Reviews in Food Science & Nutrition 54(9): 1103-1116. | Not primary research |
| Neckerman, K. M. (2014). "Takeaway food and health : Change the menu, not the venue." BMJ (Online) 348(g1817). | Not primary research |
| Nevarez, C. R., et al. (2013). "Salud Tiene Sabor: A Model for Healthier Restaurants in a Latino Community." *American Journal of Preventive Medicine* 44(3 supplement 3): S186-S192. | No before/after measures |
| Nikolaou, C. K., et al. (2015). "Calorie-labelling: Does it impact on calorie purchase in catering outlets and the views of young adults?" International Journal of Obesity 39(3): 542-545. | Not primary research |
| Nikolaou, C. K., et al. (2015). "The effect of calorie-labelling of meals on macro- and micronutrients chosen by young-adults over 9-months." Obesity Facts 8: 46-47. | Setting not in included OHFO |
| Nordström, J. and L. Thunström (2015). "The impact of price reductions on individuals’ choice of healthy meals away from home." Appetite 89: 103-111. | Setting not in included OHFO |
| Nothwehr, F. L. Snetselaar, J. Dawson, C. Hradek and M. Sepulveda (2010). "Healthy option preferences of rural restaurant customers." *Health Promotion Practice* 11(6): 828-836. | No OHFO intervention e.g. survey data |
| Nothwehr, F., et al. (2014). "Statewide dissemination of a rural, non-chain restaurant intervention: adoption, implementation and maintenance." Health Education Research 29(3): 433-441. | No outcomes reported or not outcome of interest |
| Okie, S. (2007). "New York to trans fats: you're out!" *New England Journal of Medicine* 356(20): 2017-2021. | Not primary research |
| Pande, A. H., et al. (2011). "Calorie labelling on High street: Serious methodological flaws compromise study findings.” *British Medical Journal* 343(7823): 549-550. | Not primary research |
| Papies, E.K., and H. Veling. (2013). “Healthy dining. Subtle diet reminders at the point of purchase increase low-calorie food choices among both chronic and current dieters.” *Appetite* 61:1-7. | No before/after measures |
| Paradis, G., J. O'Loughlin, M. Elliott, P. Masson, L. Renaud, G. Sacks-Silver and G. Lampron (1995). "Coeur en santé St-Henri--a heart health promotion programme in a low income, low education neighbourhood in Montreal, Canada: theoretical model and early field experience." *Journal of Epidemiology and Community Health* 49(5): 503-512. | No before/after measures |
| Rajczi, A. (2013). "Formulating and articulating public health policies: The case of new york city." Public Health Ethics 6(3): 246-251. | Not primary research |
| Rendell, S. L. (2014). Availability of point-of-purchase calorie labeling: Its relationship to food purchasing decisions. US, ProQuest Information & Learning. 74. | No before/after measures |
| Rendell, S. L. and C. Swencionis (2014). "Point-of-Purchase Calorie Labeling Has Little Influence on Calories Ordered Regardless of Body Mass Index." Current Obesity Reports 3(3): 368-375. | No before/after measures |
| Richard, L. J. O’loughlin, M. Masson, L. and S. Devost (1999). "Healthy Menu Intervention in Restaurants in Low-Income Neighbourhoods: A Field Experience." *Journal of Nutrition Education* 31(1): 54-59. | No before/after measures |
| Ross, G. L. (2010). "Determining the benefits of the New York City trans fat ban." *Annals of Internal Medicine* 152(3): 194-195. | Not primary research |
| Salvador, G. Miranda, G. Castell, C. Cabezas, C. and A. Mateu (2013). “The Amed certification enables to follow a healthy eating diet outside the house in Catalonia for more than 38,000 people/day.” *Annals of Nutrition and Metabolism* 62: 64. | Setting not in included OHFO |
| Sarink, D., et al. (2014). "Calorie labeling on menus and menu boards." Jama 312(24): 2688-2689. | Not primary research |
| Schwartz, J., et al. (2012). "Inviting Consumers To Downsize Fast-Food Portions Significantly Reduces Calorie Consumption." *Health Affairs* 31(2): 399-407. | Setting not in included OHFO |
| Shovelin, K. M. Molloy, D. Yum, K. Shirah, K. Andersen, J. Ezzell and D. Beth (2003). Changing the environment in which consumers dine: The Winner's Circle Healthy Dining Program. *The 131st Annual Meeting of APHA.* | No before/after measures |
| Sinclair, S. E., et al. (2014). "The influence of menu labeling on calories selected or consumed: a systematic review and meta-analysis." Journal of the Academy of Nutrition and Dietetics 114(9): 1375-1388.e1315. | Not primary research |
| Sosa, E. T., et al. (2014). "Associations Between a Voluntary Restaurant Menu Designation Initiative and Patron Purchasing Behavior." Health Promotion Practice 15(2): 281-287. | No before/after measures |
| Stein, J. (2011). "Putting the facts on menus." *Los Angeles Times -- Southern California Edition (Front Page)* E4-E4. | Unable to find full text |
| Stein, K. (2011). "A National Approach to Restaurant Menu Labeling: The Patient Protection and Affordable Health Care Act, Section 4205." *Journal of the American Dietetic Association* 111(5): S19-27. | Not primary research |
| Stran, K. A., et al. (2013). "Mandating nutrient menu labeling in restaurants: potential public health benefits." The Journal of the Arkansas Medical Society 109(10): 209-211. | Not primary research |
| Sturm, R. and D. A. Cohen (2009). "Zoning for health? The year-old ban on new fast-food restaurants in South LA." *Health Affairs (Project Hope)* 28(6): 1088-1097. | No before/after measures |
| Tan, A. S. L. (2009). "A case study of the New York City trans-fat story for international application." *Journal of Public Health Policy* 30(1): 3-16. | No outcomes reported or not outcome of interest |
| Tandon, P.S. Wright, J. Zhou, C. Rogers, C.B. and D.A. Christakis (2010). “Nutrition Menu Labeling May Lead to Lower-Calorie Restaurant Meal Choices for Children.” *Pediatrics* 125: 224 | Setting not in included OHFO |
| Thornton, L. E., et al. (2013). "Barriers to avoiding fast-food consumption in an environment supportive of unhealthy eating." Public Health Nutrition 16(12): 2105-2113. | No before/after measures |
| Timmerman, G. M. and A. Brown (2012). "The Effect of a Mindful Restaurant Eating Intervention on Weight Management in Women." *Journal of Nutrition Education & Behavior* 44(1): 22-28. | Setting not in included OHFO |
| Vermeer, W. M., et al (2014). "Small, medium, large or supersize? The development and evaluation of interventions targeted at portion size." International journal of obesity (2005) 38: S13-S18. | Not primary research |
| Wang, Y. C. and S. M. Vine (2013). "Caloric effect of a 16-ounce (473-mL) portion-size cap on sugar-sweetened beverages served in restaurants." *American Journal of Clinical Nutrition* 98(2): 430-435. | No OHFO intervention e.g. survey data |
| Wansink, B. and D. Just (2012). "The Limits of Changing Defaults in Fast-Food Restaurants and the Surprising Solution for a Better Happy Meal." *Journal of Nutrition Education & Behavior* 44(4S1): S62-S62. | Setting not in included OHFO |
| Webb, R. (2003). "Healthy eating. A chef's secret. Fast food done right." *Diabetes Forecast* 56(12): 31-33. | Unable to find full text |
| Wellard, L., et al. (2012). "Sales of healthy choices at fast food restaurants in Australia." *Health Promotion Journal of Australia* 23(1): 37-41. | No OHFO intervention e.g. survey data |
| Wisdom, J., J. S. Downs and G. Loewenstein (2010). "Promoting Healthy Choices: Information versus Convenience." American Economic Journal: Applied Economics 2(2): 164-178. | No before/after measures |
| Wootan, M. G. (2007). "Need for and Effectiveness of Menu Labeling." *Journal of the American Dietetic Association* 107(1): 33-34. | Not primary research |
| Yan Chan, E. K. and B. Wansink (2011). "McHealthy: How frequent dining program at fast food restaurant increases healthy eating intention." *FASEB Journal* 25. | No before/after measures |
| Yoon, H. J. (2010). “Consumers' food choice at a restaurant depending on nutritional information and nutritional menu context.” *US, ProQuest Information & Learning*. 70. | Unable to find full text |
