# Supplementary material for: The impact of interventions to promote healthier ready‐to‐eat meals (to eat in, to take away or to be delivered) sold by specific food outlets open to the general public: a systematic review
Source: Obes Rev. 2016 Nov 29;18(2):227–46. doi: 10.1111/obr.12479 (PMC5244662; doi:10.1111/obr.12479)
Supplement: Supplementary file 3 — Supporting info item [file OBR-18-227-s003.docx]

## Table S3 Study characteristics

| Study ID | Design | Aim | Population | Sampling method, response rate | Sample size (approached,  baseline,  final) | Time from baseline to last follow-up | Data collection methods | Role of funding source |
| --- | --- | --- | --- | --- | --- | --- | --- | --- |
| Acharya 2006 | Repeat cross-sectional with control | To evaluate a restaurant nutrition campaign to promote healthy main meal choices | 50% F; adult; 76% Caucasian, 11% Hispanic, 6% Asian, 4% Black;  Modal age 30 to 50 years.  Average annual household income >$69,000.  60% participants dined at that restaurant  3 to 10 times per month | Every 2nd to 5th customer was approached midweek to take part, depending on turnover,  63% response rate | 1885 approached, 1184 final | Surveys used in this analysis were conducted in May and June 2001. Baseline, preintervention surveys conducted in January 2000 | Self-administered survey | California Department of Health Services’ Cancer Prevention and  Nutrition Section, Grant #98-16024 |
| Angell 2012 | Repeat cross-sectional | To assess the effect of NYC regulation on trans and saturated fat content of fast-food purchases | 50% F; adult | Randomly sampled 300 restaurants from all 1625 NYC licensed restaurants from 13 chains which represented almost 90% of all eligible  restaurants (chain restaurants that had >15 locations nationally, had available nutritional information, and were not ice cream chains) | 300 restaurants approached, 168 analysed; The study required at least  3600 people per year for an 80% power to detect a 5%  change in calories | 9 weeks March to June 2007, 9 weeks March to June 2009 | Receipts pre and post regulation matched to available nutritional information plus brief surveys of lunchtime customers | Data collection  funded by the City of New York and Robert Wood  Johnson Foundation Healthy Eating Research Program (grant No 65839). Staff supervising  and analysing the study were employees  of the NYC Department of Health and Mental  Hygiene. |
| Bagwell 2014 | Cohort | To report findings from two evaluations of the Chartered Institute of Environmental Health’s Healthier Catering Commitment (HCC) scheme | N/R | All boroughs known to be operating the HCC scheme were invited to provide data on each of the businesses in their area that had been awarded the HCC | Total number of business eligible (awarded HCC) not reported.  Final sample = 77 businesses with usable data | N/R | Online survey completed by those directly involved in advising businesses | Greater London Authority, Chartered Institute of Environmental Health and Inner North West London Primary Care Trusts |
| Bedard & Kuhn 2013 | Repeat cross-sectional with control (econometric) – some customers were repeat customers | To assess the effects of a receipt-based intervention (Nutricate) in a chain restaurant (Burgerville) | N/R | N/R | 4875 observations | 125 weeks  Weekly purchase information for 125-week period January 2008 to mid-May 2010. Beginning on June 4, 2009, the receipts at a single store were changed to the *Nutricate* receipt | Weekly sales data | NIH grants R21 DK075642 and 3R21DK075642-02S1 |
| Bollinger 2011 | Repeat cross-sectional with control plus subgroup cohort (econometric) | To assess the impact of calorie labelling in a chain restaurant (Starbucks) | Starbucks cardholders (inside and outside of NYC) | All Starbucks restaurants in NYC, Boston and Philadelphia | 222 Starbucks locations in NYC, 94 Starbucks  locations in Boston and Philadelphia >100 million transactions;  cardholder sample averaged at least one transaction per week;  = 7,520  individuals in NYC and 3,772 individuals in Boston and Philadelphia, (1.51 million transactions) | 14 months: every transaction January 2008 to February 2009 (mandatory calorie posting introduced April 2008) | All till transactions, cardholders transactions, in-store customer surveys | Authors state ‘no consulting relationship with Starbucks—the  findings in this study are completely independent of Starbucks’ interests’ |
| Bruemmer 2012 | Cohort | To examine if following implementation of King County menu labelling regulation a) individual menu items were reformulated to improve nutrition profile and b) nutrient profiles of overall menu had been reformulated | NR | Purposive sampling of chains meeting criteria. Paper reports convenience sampling of restaurants within chains | 37 of 92 total regulated chains (one restaurant audited per chain) | 2 time points: 6-months and 18-months post legislation, May to July 2009 and May to July 2010; same restaurants audited at each time point except 1 | Audit of menu items by study personnel. Collected in restaurant or from the chains website. Study staff trained on data collection by first author. | Grant nos. 67291 and 65233 from Healthy Eating Research program of the Robert Wood Johnson Foundation |
| Chen 2015 | Repeat cross sectional (2008, 2009, and 2010) | To examine if, following implementation of King County menu labelling regulation, calorie information awareness and use increased. | Respondents of the Washington State Behavioural Risk Factor Surveillance System (BRFSS) landline telephone survey, English speaking and 18 years or older, living in King County. | All those who reported eating at a regulated chain in the past week (3132 of 8737) | 3132  (796 in 2008, 979 in 2009, and 1086 in 2010) | 2 years; 2008 to 2010 | Data used were answers to two questions in the BRFSS survey. | Grant nos. 67291 and 65233 from Healthy Eating Research program of the Robert Wood Johnson Foundation |
| Downs 2013 | Controlled clinical trial | To assess the additional effect of adding calorie recommendations to calorie labelling on chain restaurant menus | 53% F; mean age 36 years, range 18-89 years; 36% African American; 16% White; 14% Hispanic; 26% N/R | All adults entering the restaurants at lunchtime were invited to take part, over two thirds of recommendation slips were returned | Baseline: 624;  Final: 497; | 2-4 months 2 months prior  to and following implementation of menu labelling  regulations in 2008,3 subsets randomised at each timepoint (different participants pre and post labelling) | Receipts, exit surveys | US Department  of Agriculture Economic Research Service (grants  58400060114 and 59400080077) and a grant  from the Center for Behavioral Decision Research,  Carnegie Mellon University |
| Dumanovsky 2011 | Repeat cross-sectional | To assess NYC calorie-labelling regulation on energy content  . | 50% F; adult | Randomly sampled 300 restaurants from all 1625 NYC licensed restaurants from 13 chains which represented almost 90% of all eligible  restaurants (chain restaurants that had >15 locations nationally, had available nutritional information, and were not ice cream chains) | 7750 receipts at baseline, 8730 at follow-up, 7311 receipts at baseline and 8489 receipts at follow-up;  The study required at least  3600 people per year for an 80% power to detect a 5%  change in calories | 9 weeks March to June 2007, 9 weeks March to June 2009 | Receipts pre and post regulation matched to available nutritional information plus brief surveys of lunchtime customers | Data collection  funded by the City of New York and Robert Wood  Johnson Foundation Healthy Eating Research Program (grant No 65839).  All research was conducted independently of the external funder |
| Elbel 2009 | Repeat cross-sectional with control | To examine the influence of calorie labels on menus on low-income consumers' fast food choices | Children/adolescents 13-17 years: 54%; 53% F; 66% Black, 24% Latino, 11% White/other;  Adult (>17 years): mean age 38 years; 62% F; 65.7% Black, 19.9% Latino, 14.4% White/other or mixed, almost half post-labelling sample had a high school diploma or less | Street intercept survey,  response rate N/R | Youth: 427 sampled, 349 final;  Adults: 1177 sampled, 1156 final | 4 weeks: two-week period beginning 8 July 2008 repeated 4-weeks post labelling law | Receipts plus brief consumer exit survey | Robert Wood Johnson Foundation Healthy Eating Research program,  Yale Rudd Center for Food Policy and Obesity, the NYU Wagner Dean’s Fund and the National Heart, Lung,  Blood Institute (R01HL095935) |
| Elbel 2013 | Repeat cross-sectional (pre and post legislation) with control cohort *(difference in difference design)* | To determine whether nutrition labelling on restaurant menus resulted in a lower number of calories purchased by adults | Adults only; mean age 39 years. Gender 54% men, 7% missing; 70% Black, 20% White, 10% Latino/other, 61% high school education or less, 3% missing. | Approached and recruited as diners entered the restaurant. Diners were offered $5 if they were willing to give their receipt to the researchers on exit, and answered a few questions. | Unclear how many people were approached. Full sample = 2083. Baseline intervention 599, final 570; baseline control 433, final 481. | One year; 2009 - 2010 | Till receipts | NIH/NHLBI grant No R01HL095985 |
| Eldridge 1997 | Repeat cross-sectional | To develop healthier meal choices for patrons of the Target shopping stores through low-fat and calorie labelling | Market is young, well-educated, moderate-to-better-income families living active lifestyles. Median age of shoppers is 42 years; median household income $34,000 per year; | 7 Target stores were selected because (1) all had similar menus, (2) preparation of menu items was identical at each site (3) all used the same computerized  checkout system  . | N/R | 12 months, 4 timepoints, quarterly trends  of 'Good for You’ items as a percentage of total sales by food  category.1-3 months prior, 13 months post, 4-6 months post, 7-9 months post | Sales data from the computerised check out system; produced monthly sales totals of each  menu item on summary register tapes | NR |
| Finkelstein 2011 | Repeat cross-sectional with control (econometric) | To examine the effect of mandatory menu-labelling regulation  In one Mexican fast-food chain (Taco Time) | N/R | Taco Time was the only fast food chain that agreed to provide transaction data.  Random selection of subset of intervention restaurants (control restaurants from adjacent counties) | Data provides >80% power to detect differences  of 25 calories or more per monthly transaction | 2 years, total monthly transactions and  monthly sales for every menu item between January 2008 and  January 2010 | Total monthly transactions and monthly sales for every menu item. Calorie data from company website | internal grant from Duke-  NUS Graduate Medical School |
| Fitzgerald 2004 | Repeat cross-sectional | To determine if a promotional campaign impacted on the sales of heart-healthy menu items at community restaurants | N/R | Restaurants were eligible to participate  if they were current participants in the ‘Healthy Dining Progam’ (HDP)  and used electronic cash registers | N/R | 4-week baseline tracking period,  8-week promotional campaign (January 2001 to February 2001), 4-week post-campaign  tracking period | Weekly tracking logs of sales of HDP items using electronic cash register receipts | NR |
| Gase 2015 | Cohort | To examine the programmatic impact on menu items and children’s meal offerings at retail restaurants that participated in LA County’s voluntary public recognition program, *Choose Health LA Restaurants* | Licensed retail restaurants that had successfully applied to the recognition program between July 2013 and 2014 | All eligible restaurants were included in the study, 100% response rate | 17 restaurant brands (42 restaurant locations), no drop-outs between baseline and follow-up | NR | Menus were analysed using a standardised assessment form | Cooperative agreement form the Centers for Disease Control and Prevention (#1U58DP003631-01) and grant agreement fom First 5 LA (#08379.1) |
| Hanni 2009 | Cohort | To increase and promote healthier menu options in taquerias; to encourage healthier menu choices by clientele; to develop local business owners as agents of health-related change | 64% Hispanic of which 88% Mexican descent; young, low education levels,  spoke Spanish at home, lived in USA 10 years or more | Purposive, all 35  taquerias selected from low-income neighbourhoods for baseline survey | 35 restaurants approached for baseline survey, 16 approached for intervention | 3 year evaluation (midpoint) plus case study | Survey administered by health department survey workers | cooperative agreement with  the Centers for Disease Control and Prevention |
| Horgen & Brownell 2002 | Controlled clinical trial | To test the effects on purchasing of health information and pricing interventions | 225 to 275 patrons/day; Caucasian; upper-middle-class. 6% response rate to survey for demographic information | Purposive, restaurant was chosen because healthy items were available  on the menu and the restaurant had run price specials previously | 101 data points for each  food item (n=8) | 101 days of data collection across six  intervention periods | Electronic till register | NR |
| Krieger 2013 | Repeat cross-sectional (retrospective) | To examine the effect of menu labelling on calories purchased, and secondarily, to assess self-reported awareness and use of labels | English speaker, aged at least 14 years, with itemised receipt.  59% male, 76% white non-Hispanic, 53% aged <40 years. | Stratified, two-stage cluster random sampling of  10 most common fast-food chains;  sampling  strategy resulted in a diverse set of chains not dominated by chains with  the most locations in the county | 90% approached,  57% of all eligible customers participated,  7325 customers pooled across all time points | 3 timepoints: baseline (1–3 months prior to regulation implementation); Post 1 (4–6 months after); and Post 2 (16–18 months after) | Receipt and exit survey | Healthy Eating Research (Grants  65233 and 67291), a national program of the Robert Wood  Johnson Foundation |
| Lee-Kwan 2013 | Controlled clinical trial | To evaluate promotion of healthy options in prepared food sources in low-income urban settings | Low-income African-American adults who visited carryout more than once per month in previous year | Stratified random selection of carry-outs by geographic location and race/ethnicity of storeowners (Korean-Americans and African-Americans).  66% response rate | 101 for intervention exposure survey. 3552 total main course sales in intervention sites. | Receipts collected weekly and total sales were tracked for all carry-outs; 10 store visit evaluations; intervention exposure survey at end of intervention (every fifth customer for 1.5hrs/day over 10 days per store). Formative research April 2009 to December 2010, development of intervention December 2010 to April 2011, implementation of pilot study February 2011 to September 2011 (7-months) | Receipts, carry-out visits and exit survey. | Diabetes Research & Training Center, Johns Hopkins & University of Maryland, and the Center for a Livable Future (Eating for the  Future Fellowship & Carl Taylor Research Grant Award), Johns Hopkins University |
| Licata 2002 | Repeat cross-sectional plus subgroup cohort | To measure whether rates of health promotion practices among restaurants and cafes in one region of Australia increased as a result of telemarketing intervention | N/R | All restaurants and cafes in local directory selected,  91% (321) restaurants and cafes participated at baseline and 81% (239) at follow-up; 122 restaurants and cafes participated in both | 352 approached,  321 baseline, 239 final, 122 in cohort (restaurants and cafes) | 3 years, 2 timepoints, 1997 and 2000 after maximum 3 annual telemarketing calls | Telephone interviews of licensees or managers | NR |
| Namba 2013 | Repeat cross-sectional with control (retrospective)  Case-control restaurants | To assess the impact of nutrition labelling policies on menu offerings in large chain fast food restaurant | N/R | All large restaurant chains (20 or more locations nationally) that posted nutrition information on their website for at least 6 years (not coffee shops) | Only 9 out of 50 chains met eligibility criteria.  4,055 menu items, final sample 3887 items: 2529 adult a la carte main meals, 1186 adult sides, 172 children’s a la carte main meals | 7 years, 2005 to 2011 | Compared menus, retrospective nutrient analysis of publically accessible restaurant websites | Drexel University and externally from National Institutes for Health, Eunice Kennedy Shriver National Institute of Child Health and Human Development under grant number U01-HD061978 |
| Nothwehr 2013 | Repeat cross-sectional. Customers were eligible to participate at all points if present in the restaurant | To influence restaurant customer ordering of healthier choices by point of entry and table signs | 59% F; mean age 53 years, 98% white | Non-chain (owner-operated) restaurants identified by research staff familiar with nearby towns, each in a small rural town with no overlap in customers,  all owners who were approached agreed to participate.  Researcher  approached all customers appearing 18+ to invite to participate in self-administered anonymous survey | The a  priori sample size goal of 70 customers per restaurant  per time point was reached  (α = .05, 80% power to detect longitudinal differences  in key variables).  At 3, 6 and 12-months, the number of order slips  collected (1-week periods) ranged from 506 to 1,938 for each restaurant. Response rate was 70% to 84%  Baseline: 363;  3-months: 370;  6-months: 157; 12-months: 141; | 12-months, baseline data collection  in 3 restaurants in June 2007. One  of these restaurants closed  and was replaced by 4^th^ restaurant with baseline  data collection June 2008.  Follow-up  data collection,  October (Time 2),  February (Time 3), and June (Time 4). The replacement  restaurant dropped out of the study after Time 2  because the owner left town and the temporary manager  was unsupportive of the study. Thus, there are  data for four restaurants at baseline and Time 2 and two  restaurants at Times 3 and 4. | Brief self-administered anonymous survey, order slips collected, interview with owners; all at 3, 6 and 12-months | National Heart,  Lung, and Blood Institute, National Institutes of Health, Grant  Number 1R21HL087761-01 |
| Pandya 2013 | Repeat cross-sectional | To assess the effects of a Healthy Restaurant Intervention on % customers’ purchases of healthier main meal options in two Latino family-owned restaurants | Low-income predominantly Latino neighbourhood | Recipients of ‘Healthy Restaurant Award’, serving traditional Latino food (burritos, tacos, enchiladas); with electronic receipt counter, located in zip code 66101 and Latino family-owned | 740 total items purchased during baseline;  1429 total items purchased during intervention | 17-18 weeks, data collected 1 day per week | Electronic receipts and corresponding handbills; self-reported modifications by owners | NR |
| Pulos & Leng 2010 | Repeat cross-sectional | To assess the impact of voluntary menu labelling of 'regular' menu items on the nutritional composition of main meal purchases in full service restaurants | 55% F; 17% aged 18-30 years; 31% aged 31-45; 40% aged 46-64; 12% aged 65 or older | 6 restaurants recruited ‘in a variety of ways’ from 600 locally owned restaurants, restaurants received publicity as incentive. Survey offered to all adult diners within 2-hour sampling frame post labelling intervention, response rate 51% to 87% | 16,000 main meals purchased; 206 survey respondents | Sales data 30 days before and 30 days after menu change. Restaurants surveyed Autumn 2008 and winter of 2009. 2 of 6 restaurants were  surveyed almost a year after menus labelled; other restaurants were surveyed less than 2 months after menus labelled | Itemised sales data of main meals that were labelled with nutritional information provided by restaurants;  Customer survey | NR |
| Reimann 2015 | Cohort | To investigate whether adult “full-size portion customers” could be incentivised to eat less | Customers who had planned to eat a full-sized sandwich; 39% F; mean age 29.1 years | Customers were approached as they began their order | 565 customers | Very brief intervention (minutes) – time taken to decide whether to choose half portion with lottery incentive | Question asking customers their menu choice (before intervention), observed sale (post-intervention) | National Cancer Institute Grant R01CA152062 and Joseph Newman Young Scholar’s Fund of the University of Arizona |
| Saelens 2012 | Controlled before and after study (retrospective) | To examine changes in restaurant environments from before to after nutrition labelling | Half of restaurants were located in lower-income and more racially/ethnically diverse area | Fast food restaurants from the top 10 chains were selected within each county from  among chains subject to nutritional labelling regulation (or as proposed in Multnomah  County) | 49 King County restaurants and 49 Multnomah restaurants | 3 time points, In King County, Wave 1 (pre-regulation) was conducted in October–December,  2008, Wave 2 in April–May 2009, and Wave 3 in May–June 2010. Multnomah County  restaurants were evaluated for Wave 1 in May–June 2009 and Wave 2 in November–  December 2009 | ‘Nutrition Environment Measures Surveys–Restaurant version’; permission not sought from the restaurants to conduct  the audit and restaurants not told when visit would occur | Robert Wood Johnson Healthy Eating Research Program Grants 65233, 65430,  67291 and NIH/National Institute of Environmental Health Sciences ES014240 |
| Shah 2014 | Controlled clinical trial  Four menu arms: unhealthy label, surcharge on unhealthy food (sin tax) , and both, and control | To determine whether nutrition labelling, a surcharge on unhealthy dishes (sin tax), or both, had an impact on the number of unhealthy main dishes ordered by men and women. | Diners at the restaurant | Unclear how the researchers selected the one restaurant used in the study. | It appears that researchers did not ask diners for consent and food orders from all ‘tables’ on the study dates were assessed.  464 tables, 1.063 people (63% women). | N/A  Trial tool place over a 16-day period (Mon-Thur, on 4 consecutive weeks). A different menu was used on each day, each week, counterbalancing across days of the week. | Menu orders collected by the waiting staff. | Unclear but 1^st^ author was a doctoral student at the time of writing the paper. |
| Tandon 2011 | Controlled before and after study (same participants) retrospective | To determine whether nutrition labelling on restaurant menus resulted in a lower number of calories purchased by children and their parents | Children aged 6-11 years: 49% F; mean age 9 years; 14% Hispanic in San Diego County; 1% Seattle/King County; 70% parents had college degree or higher; more families had higher incomes in Seattle/King County than San Diego County (70% vs 39% >$90,000). Sixty-four percent of parents and 25% of children were overweight/obese. | Recruited from the Neighborhood Impact on Kids Study; English-speaking parents indicating their child ate at a fast-food chain subject to the Seattle/King County  menu-labelling regulation were eligible | 251 approached,  145 enrolled,  133 children and their parents in final analysis.  A sample size of 75 child–parent pairs in each  county had 80% power to detect a 100-calorie difference in calories purchased across  counties with a two-sided *t*-test (SD=220, alpha=0.05). | Parents were  instructed to go to the restaurant with their child before January 1, 2009 (the date of labelling  implementation), process was repeated post-regulation  March –June 2009. | Respondents would mail back receipt. Via telephone survey, receipt items were clarified and information on meal selection and nutrition information awareness collected; calorie information from companies websites | NIH (Neighborhood  Impact on Kids, ES014240), the Robert Wood Johnson Foundation (#65233) Healthy Eating Research National  Program, and Seattle Children's Hospital Research Institute |
| Wansink 2014 | Repeat cross-sectional | To study changes in purchasing patterns of meal components (in response to changing child meal bundle options) to understand the degree to which within-meal calorie compensation might have occurred | Data from anonymous transaction records from 30 McDonalds restaurants (demographically representative of the Unites States) | ‘Representative’ restaurants of the United States (sampling method not reported so unsure how the 30 restaurants were selected). All the restaurants fit three specific criteria: 1) transaction  data were collected during the full study period; 2) the restaurants  were geographically dispersed but demographically representative of the  United States; and 3) the general sales patterns from the restaurants were  similar to national averages | Sample sizes not reported | Data collected in June, July and August 2011 (pre-changes) and 2012 (post-changes) | Anonymous transaction records | Project was partially funded by a grant from McDonald’s Corporation |
| Wiggers 2001 | Repeat cross-sectional plus subgroup cohort | To measure whether rates of health promotion practices among licensed clubs and hotels in one region of Australia increased as a result of telemarketing intervention | N/R | All hotels, registered clubs and nightclubs in Hunters Valley, 93% response rate | 333 outlets approached;  311 at baseline;  201 at final | 3 years, 2 timepoints, 1997 and 2000 after maximum 3 annual telemarketing calls | Telephone interviews of licensees or managers; 25 randomly selected premises were visited to confirm validity of survey | NR |

**Key:** F: female; N/A: not applicable; NR: not reported; NYC: New York City
