# Supplementary material for: The impact of interventions to promote healthier ready‐to‐eat meals (to eat in, to take away or to be delivered) sold by specific food outlets open to the general public: a systematic review
Source: Obes Rev. 2016 Nov 29;18(2):227–46. doi: 10.1111/obr.12479 (PMC5244662; doi:10.1111/obr.12479)
Supplement: Supplementary file 4 — Supporting info item [file OBR-18-227-s004.docx]

Table S4 Quality Assessment of included studies

| Study | A: Selection bias | B: Study design* | C: Confounders | D: Blinding | E: Data collection methods | F: Withdrawals and drops-outs | Global rating |
| --- | --- | --- | --- | --- | --- | --- | --- |
| Acharya 2006 | Moderate | Moderate | Moderate | Weak | Moderate | NA | Moderate |
| Angell 2012 | Moderate | Weak | Strong | Moderate | Strong | NA | Moderate |
| Bagwell 2014 | Weak | Moderate | Weak | Weak | Weak | Weak | Weak |
| Bedard & Kuhn 2013 | Weak | Moderate | Moderate | Weak | Strong | NA | Weak |
| Bollinger 2011 | Moderate | Strong | Moderate | Moderate | Strong | NA | Strong |
| Bruemmer 2012 | Weak | Moderate | Weak | Moderate | Weak | NA | Weak |
| Chen 2015 | Moderate | Weak | Moderate | Weak | Weak | NA | Weak |
| Downs 2013 | Strong | Strong | Strong | Weak | Strong | NA | Moderate |
| Dumanovsky 2011 | Moderate | Weak | Strong | Moderate | Strong | NA | Moderate |
| Elbel 2009 | Weak | Moderate | Moderate | Moderate | Strong | NA | Moderate |
| Elbel 2013 | Moderate | Moderate | Moderate | Moderate | Strong | NA | Moderate |
| Eldridge 1997 | Moderate | Weak | Weak | Moderate | Strong | NA | Weak |
| Finkelstein 2011 | Moderate | Moderate | Weak | Moderate | Strong | NA | Moderate |
| Fitzgerald 2004 | Moderate | Weak | Weak | Moderate | Strong | NA | Weak |
| Gase 2015 | Strong | Moderate | Weak | Weak | Strong | Strong | Weak |
| Hanni 2009 | Moderate | Moderate | Weak | Weak | Weak | Weak | Weak |
| Horgen & Brownell 2002 | Weak | Strong | Weak | Moderate | Strong | NA | Weak |
| Krieger 2013 | Moderate | Weak | Moderate | Moderate | Strong | NA | Moderate |
| Lee-Kwan 2013a | Moderate | Strong | Weak | Strong | Strong | NA | Moderate |
| Licata 2002 | Moderate | Moderate | Weak | Moderate | Weak | NA | Weak |
| Namba 2013 | Moderate | Moderate | Moderate | Moderate | Moderate | NA | Strong |
| Nothwehr 2012 | Strong | Weak | Weak | Moderate | Strong | NA | Weak |
| Pandya 2013 | Weak | Weak | Weak | Moderate | Strong | NA | Weak |
| Pulos & Leng 2010 | Weak | Weak | Moderate | Moderate | Moderate | NA | Weak |
| Reimann 2015 | Moderate | Moderate | Weak | Moderate | Strong | NA | Moderate |
| Saelens 2012 | Moderate | Strong | Moderate | Moderate | Strong | NA | Strong |
| Shah 2014 | Strong | Strong | Moderate | Moderate | Strong | NA | Strong |
| Tandon 2011 | Weak | Strong | Moderate | Weak | Strong | Strong | Weak |
| Wansink 2014 | Moderate | Weak | Weak | Moderate | Strong | NA | Weak |
| Wiggers 2001 | Moderate | Moderate | Weak | Moderate | Weak | NA | Weak |

*see Box 1 Typology of study designs
