# Supplementary material for: The impact of interventions to promote healthier ready‐to‐eat meals (to eat in, to take away or to be delivered) sold by specific food outlets open to the general public: a systematic review
Source: Obes Rev. 2016 Nov 29;18(2):227–46. doi: 10.1111/obr.12479 (PMC5244662; doi:10.1111/obr.12479)
Supplement: Supplementary file 5 — Supporting info item [file OBR-18-227-s005.docx]

## Table S5 Interventions

| Study ID | Design | OHFO type | Intervention description | Nuffield Intervention Ladder Code | Theoretical Framework | Behaviour Change Wheel | |
| --- | --- | --- | --- | --- | --- | --- | --- |
|  |  |  |  |  |  | Intervention Function | Policy category |
| Acharya 2006 | Repeat cross-sectional with control | 4 restaurant chains, California, USA | Multifaceted social marketing campaign to promote healthy main meals ‘TrEAT Yourself Well Restaurant Nutrition Campaign’ that included in-restaurant promotions (table tents, posters, etc.), community events (seminars, food tasting, educational programs), information distributed by community health professionals (dietitians, physicians, health educators, etc.), television advertising, and magazine advertisements. Additional promotional activities included waitstaff incentives, custom newspaper inserts, direct mailing, distribution of food samples at neighbourhood apartments and businesses, and frequency/discount cards. $5.00 gift certificate was offered to each person who completed survey | Enable choice | Consumer behaviour model based on the Theory of Reasoned action | Environmental restructuring; Education;  Incentives;  Persuasion | Communication/marketing;  Environmental/social planning |
| Angell 2012 | Repeat cross-sectional | 168 restaurants from 11 fast-food chains, NYC, USA | NYC regulation on the trans and saturated fat content of fast-food purchases: restricts all food service establishments, including both chain and non-chain restaurants, from using, storing, or serving food that contains partially hydrogenated vegetable oil and has a total of 0.5 g or more trans-fat per serving. $2 Metrocard was offered as an incentive | Restrict choice | NR | Environmental restructuring | Environmental/social planning; Legislation |
| Bagwell 2014 | Cohort | 77 small independent catering outlets, UK | The scheme is designed to be implemented by EHOs, although in some boroughs, support from nutritionists and other public health professionals is also available.  To gain the Award, businesses need to meet a minimum of eight criteria (from a list of 22) that include conditions in relation to the use of fats and oils, salt, sugar, milk and spreads, fruit and vegetables, portion size and promotion of healthier options. Four of the criteria, concerning the type of oil used, use of salt, availability of diet drinks, and smaller portions, are essential criteria that all businesses must comply with. A further three concern the maintenance of cooking oil and are mandatory criteria for those involved in deep-fat frying. | Restrict choice | NR | Restriction; Environmental restructuring; Education | Communication/marketing; Regulation; Environmental/social planning |
| Bedard & Kuhn 2013 | Repeat cross-sectional with control | 39 Burgerville restaurants, California, USA | Nutricate receipt intervention in one of 39 restaurants - receipts designed by *SmartReceipt* Corporation included personalised suggestions designed to reduce fat and calorie consumption.  *Nutricate* receipt consists of three components: information, motivation and recommendations. The information component displays each item’s relevant nutritional data, as ordered. Thus, if a consumer asked to "hold the mayo," on her order, the nutritional information provided on the receipt will reflect this. Receipts can also contain *motivational* statements, aimed to remind consumers of the benefits of a healthier diet, such as “A diet low in total fat may reduce the risk of heart disease.” Customized *recommendations* for how a customer can improve his/her nutrition, by making small changes to the items the customer just ordered. For example, one message used at Burgerville says, “Did you know if you held the mayonnaise on a burger you would cut your fat intake by 57%, or 15 grams?” | Provide information | NR | Environmental restructuring;  Education;  Persuasion | Communication/marketing |
| Bollinger 2011 | Repeat cross-sectional with control plus subgroup cohort | Starbucks Cafes in NYC (intervention), Boston (control) and Philadelphia (control), USA | NYC calorie labelling legislation: restaurant chains with 15 or more outlets nationally are required to clearly post calorie content of regular menu items next to the price on all menus, menu boards and item tags at point of purchase | Provide information | NR | Environmental restructuring; Education | Communication/marketing;  Environmental/social planning;  Legislation |
| Bruemmer 2012 | Cohort | Chain restaurants subject to King Country regulation with >4 establishments. Included sit down and fast food chains. Audit was limited to restaurants whose product line was  primarily burgers (eg, McDonalds, Burger King), pizza (eg,  Pizza Hut, Dominos), sandwich/sub (eg, Subway, Blimpie), or  Tex-Mex (eg, Taco Time, Taco del Mar), USA | Mandatory menu labelling in King County: requires all restaurant chains with 15 or more locations to have nutrition labels (calories, saturated fat, carbohydrates, and sodium) for all standard food and beverage items at the point of purchase. Fast food restaurants are required to display calories on menu boards or on signs adjacent to menu boards and must make information on carbohydrate, sodium, saturated fat, and daily recommended caloric intake readily available in pamphlets, brochures, or posters. Additionally, restaurants were required to post calories on drive-through menu boards | Provide information | NR | Environmental restructuring; Education | Communication/marketing;  Environmental/social planning;  Legislation |
| Chen 2015 | Repeat cross sectional | Regulated chain or fast food restaurants in King County, USA | Mandatory menu labelling in King County: requires all restaurant chains with 15 or more locations to have nutrition labels (calories, saturated fat, carbohydrates, and sodium) for all standard food and beverage items at the point of purchase. Fast food restaurants are required to display calories on menu boards or on signs adjacent to menu boards and must make information on carbohydrate, sodium, saturated fat, and daily recommended caloric intake readily available in pamphlets, brochures, or posters. Additionally, restaurants were required to post calories on drive-through menu boards | Provide information | NR | Environmental restructuring; Education | Communication/marketing;  Environmental/social planning;  Legislation |
| Downs 2013 | Controlled clinical trial. Block (2 hour blocks) randomisation to avoid contamination between customers. Two surveys pre and post labelling law (different client groups at each). | 2 McDonalds restaurants in NYC, USA | Supplementing mandatory NYC calorie labelling with calorie recommendations  3 groups: calorie posting on menus (law in 2008) + either 1) daily calorie recommendations, 2) per meal calorie recommendations, 3) no recommendations. Customers approaching the restaurant were handed a slip with 1 of 3 recommendations, and asked to retain their receipt for participation in an exit survey. 5$ for participation. | Provide information | NR | Environmental restructuring; Education | Communication/marketing;  Environmental/social planning |
| Dumanovsky 2011 | Repeat cross-sectional | 168 restaurants from 11 fast-food chains, NYC, USA | NYC calorie labelling legislation: restaurant chains with 15 or more outlets nationally are required to clearly post calorie content of regular menu items next to the price on all menus, menu boards and item tags at point of purchase.  $2 Metrocard was offered as an incentive | Provide information | NR | Environmental restructuring; Education | Communication/marketing;  Environmental/social planning;  Legislation |
| Elbel 2009 | Repeat cross-sectional with control | All restaurant chains in NYC with 15 or more establishments; comparison Newark, New Jersey (McDonald’s, Burger King, Wendy’s, KFC), USA | NYC calorie labelling legislation: restaurant chains with 15 or more outlets nationally are required to clearly post calorie content of regular menu items next to the price on all menus, menu boards and item tags at point of purchase.  Customers were asked to bring their receipts back and to answer a set of questions for compensation of $2. | Provide information | NR | Environmental restructuring; Education | Communication/marketing;  Environmental/social planning;  Legislation |
| Elbel 2013 | Repeat cross-sectional (pre and post legislation) with control cohort *(difference in difference design)* | Fast food restaurants (McDonald’s and Burger King) in Philadelphia (which implemented calorie labelling policies) and Baltimore (which did not and acted as a matched comparison city), USA | Mandatory menu labelling in Philadelphia (but not Baltimore). | Provide information | NR | Environmental restructuring; Education | Communication/marketing;  Environmental/social planning;  Legislation |
| Eldridge 1997 | Repeat cross-sectional | Food service areas of 7 Target stores in Minnesota, (large discount  department store chain), USA | Low-fat labelling intervention and "Good for you" promotion. New menu boards; each  "Good for you" item was labelled with a large green checkmark. The University of Minnesota developed its own criteria for low-fat food items on the Target menu. Table tents and tray liners were printed to emphasize and educate the consumer on the "Good for You” foods and provided information about the amount of fat and calories in each one. | Enable choice | NR | Environmental restructuring; Education | Communication/marketing;  Environmental/social planning |
| Finkelstein 2011 | Repeat cross-sectional with control | Taco Time Northwest is a Mexican fast-food restaurant chain with  more than 70 locations across the state of Washington; 7 King County restaurants and seven control locations, USA | Mandatory menu labelling in King County: requires all restaurant chains with 15 or more locations to have nutrition labels (calories, saturated fat, carbohydrates, and sodium) for all standard food and beverage items at the point of purchase. Fast food restaurants are required to display calories on menu boards or on signs adjacent to menu boards and must make information on carbohydrate, sodium, saturated fat, and daily recommended caloric intake readily available in pamphlets, brochures, or posters. Additionally, restaurants were required to post calories on drive-through menu boards | Provide information | NR | Environmental restructuring; Education | Communication/marketing;  Environmental/social planning;  Legislation |
| Fitzgerald 2004 | Repeat cross-sectional | 9 community restaurants - varied from counter service to table-service restaurant, Michigan, USA | The Healthy Dining Program (HDP) is a partnership between local restaurants and the university health care system. The HDP is one component in a web of community services (supermarket-based nutrition education, culinary classes, and physical fitness centres) offered by the university health promotion division.  8 week promotional campaign included promotional materials [print adverts, posters and table information] to increase awareness of HDP menu items on restaurant menus. Targeted at two levels; 135 000 people in community using the print media [adverts in local newspaper (7 times in 8 weeks) and three monthly family entertainment publications with events and resources ( 2 consecutive months) and targeted reach in 12 locations [nine HDP community restaurants, a fitness centre and two hospital clinics] promotional materials displayed in strategic locations [tables, cash register, entry point and toilets as well as hospital clinic waiting rooms. A feature article was also published in hospital newsletter [53 000 heart care patients].  An HDP main dish shall: (a) include two food groups from the following list: dairy products, meat and meat alternatives, fruits and vegetables, and grains; and (b) weigh at least 6 oz, including a secondary food item that weighs at least 40 g, and (c) contain no more than 12 g fat. | Enable choice | NR | Environmental restructuring;  Education;  Persuasion | Communication/marketing;  Environmental/social planning |
| Gase 2015 | Cohort | Licensed retail restaurants, USA | The Choose Health LA Restaurants program is a partnership between the Los Angeles County Department of Public Health (DPH) and local retail restaurants to promote the availability of healthier menu options. Restaurants participating in the program must meet three main criteria: 1) offer a minimum percentage of the total menu items in a reduced-size portion – required number is between 25% and 30% based on the total number of menu items offered; 2) ensure that all children’s meals meet the following: (a) include at least one serving of fruits or vegetables, (b) ensure that no more than half of the meal options contain fried-foods, and (c) ensure that any beverages included with meals be healthy (i.e., water or unflavoured milk); and 3) provide drinking water at no additional cost to customers. To participate, restaurants submit a completed application demonstrating menus that adhere to the program participation criteria. Each application is reviewed and then approved by assigned staff with expertise in the program’s administration. Technical assistance is available to all restaurants (if needed) to assist with the application process and to facilitate implementation of menu changes. Participating restaurants are recognized as partners in the program, receive an identifying window decal and in-store promotional materials, and are listed on the Choose Health LA website. | Restrict choice | NR | Restriction; Environmental restructuring | Regulation; Environmental/social planning |
| Hanni 2009 | Cohort | Taquerias (privately owned, fast-food-style Mexican restaurants), California, USA Community-based. | Taqueria intervention part of 5-year community-based intervention in Salinas, California. Tailored to the health literacy, linguistic, and cultural needs of the community.  Promotion of healthy menu options by marking  healthier items on the menu with the logo of *Steps to a Healthier Salinas* “Value It” social marketing campaign and new signage posting in the taqueria. The health educator, in collaboration with nutritionists developed a list of healthy food preparation suggestions, that  served as criteria for creating new healthier  menu items (e.g., vegetable-stuffed fish fillet, grilled fish fillet with salad greens, small burrito with grilled chicken, vegetables, and whole vs. refried beans). The suggested menu modifications were presented to taqueria owners during the nutrition counselling component of the intervention.  The methods that resulted in changes to menus were identified and packaged into a healthy nutrition tool kit by the lead health educator for dissemination. Health educator conducted routine (three times per year) follow-up visits to reinforce the nutrition messages and changes, ensure that the nutrition signage is in good shape and posted, and distribute promotional materials.  Samples of incentives—aprons with the *Steps to a* *Healthier Salinas* logo and the “Value It” logo;, copies of print media coverage of the taqueria intervention, and customer incentives such as skipping ropes to encourage exercise.  Samples of participation—a newspaper advertisement from *Steps to a Healthier Salinas’* social marketing campaign that highlighted a local taqueria owner who had adopted healthy menu options  Consumer outreach information—examples of a tip sheet on eating healthy at Mexican-style restaurants or taquerias, coupons for newspaper advertising | Enable choice | Asset-based community development approach (community members are active change agents) | Environmental restructuring;  Education;  Enablement;  Incentives;  Modelling;  Persuasion;  Training | Communication/marketing;  Environmental/social planning;  Guidelines |
| Horgen & Brownell 2002 | Controlled clinical trial | 1 delicatessen-style  Restaurant (Cafeteria) in Huntsville,  Alabama, USA | Six periods (1) an initial baseline, (2) a price decrease intervention, target items (low fat grilled chicken salad, low fat grilled chicken sandwich, low fat vegetable soup) price reduction of 20-30%. Advertised on board at restaurant entry and on chalkboard menu. Promoted as limited time price promotion.  (3) an interim baseline, (4) a health message intervention, the gain-framed message noted, “Healthy eating increases physical and  mental well-being. The Mill offers these and other healthy choices,” followed by a list of the target items. The loss-framed message stated,  “Unhealthy eating decreases physical and mental well-being. The Mill offers these and other healthy choices,” again followed by the list of target items. (5) a combination price decrease and health message intervention, and (6) a final baseline. | Price decrease only  Guide choice - incentives  Price decrease plus health promotion  Guide choice - incentives  Health promotion  Enable choice | The *matching model -* choice is based on the ratio of consumption values times the inverse of the delay ratio (  predicts that, because the interval between food choice and outcome (eating) is  short, the proximal satisfaction of a tasty meal would prevail over the distal goal of good health)  The *health belief model* -  health behaviours are enacted on the basis of perceptions of health threat and the likelihood  a behaviour will reduce the threat, which in turn is based on an assessment of the costs and benefits of undertaking the measure  (predicts that health messages would increase perceived benefits of eating healthy) | Price decrease only  Incentives  Price decrease plus health promotion  Environmental restructuring;  Education;  Enablement; Incentives; Persuasion  Health promotion only  Environmental restructuring;  Education;  Enablement;  Persuasion | Price decrease only  Fiscal  Price decrease plus health promotion  Communication/marketing;  Environmental/social planning; Fiscal  Health promotion only  Communication/marketing;  Environmental/social planning |
| Krieger 2013 | Repeat cross-sectional, retrospective | 53 restaurants from 10 chains in King County, Washington. Subway (11);  McDonald’s (6); Taco del Mar (8); Taco Time (5); Starbuck’s (5); Quizno’s (4); Tully’s (5); Jack in the Box  (4); Burger King (4); and Taco Bell (1), USA | Mandatory menu labelling in King County. In King County, chain restaurants with 15 or more sites nationally were required to post calorie information on their menus or menu boards. Information on saturated fat, carbohydrate, and sodium is also required, but not necessarily on the menu or near point of purchase. Each participant received $2 for participation. | Provide information | NR | Environmental restructuring; Education | Communication/marketing;  Environmental/social planning;  Legislation |
| Lee-Kwan 2013 | Controlled trial | 4 Carry-outs and 4 matched comparisons in Baltimore; "non-franchised small local food establishments that sell ready-to-eat food and beverages for off-premise consumption" Community-based.  USA | Phase 1: Menus redesigned to highlight healthier options. Green leafy vegetable symbol to indicate healthier foods. 3 healthier options selected by store owners for photograph promotion on menu with slogan "Try these fresh options!" Free new menu boards & posters & ~2000 paper menus.  Phase 2: Healthier side dishes (fruit cups, yoghurt, bananas, baked chips/crisps, pretzels) suggested to owners & promoted with point of purchase poster. Initial stock supply provided for free.  Phase 3: Introduced affordable healthier combo meals and improved food preparation methods, new healthier prep facilities in some cases (e.g. grill).  Carry-outs were given monetary incentives in the form of an initial stock (i.e. couple dozen of yogurt, a box of baked chips) for new promoted foods. Small monetary incentives, marketing materials, and technical assistance to motivate owners to sell healthier foods. Owners given weekly $25 gift card for 7 months as compensation for providing sales receipts, and free point-of-purchase materials including menu boards and posters and expert input on healthier food preparation methods. Subsidies were promised to carry-out owners to account for any decrease in profit they could have encountered from selling healthy combo meals for 2 months of Phase 3. For example, if a carry-out reduced the price of a healthy combo meal by $1.00 and sold 20 units, we would compensate the owners $20. To allow for healthier cooking methods, study provided the owners with an indoor grill | Enable choice | Development of intervention materials and strategies were based on Social Cognitive  Theory - emphasizes bi-directional relationship between individuals’ behaviours and their surrounding environmental factors. Social Marketing approach -  “Four Ps” Product, Price, Place, and Promotion. | Environmental restructuring; Education; Incentives | Communication/marketing;  Environmental/social planning |
| Licata 2002 | Repeat cross-sectional plus subgroup cohort | Restaurants and cafés; licenced hotels, clubs and nightclubs; in Hunter Valley, NSW region of Australia | Annual telemarketing intervention, one call each in 1997, 1998 and 1999. 18 health promotion practices (one of which included healthy food choices) under 6 broad headings [smoking, alcohol, nutrition, food safety, occupational health and safety, infectious diseases].  In 1997, restaurants and cafes were interviewed by phone to assess practice relating to each health promotion area.  Health area: Nutrition. Health Promotion Initiative: Provision of healthy food choices. Resource offered: printed information about nutrition and healthy menu guidelines | Enable choice | NR | Education | Communication/marketing;  Environmental/social planning;  Service provision |
| Namba 2013 | Repeat cross-sectional with control (retrospective)  Case-control restaurants | Large chain fast food restaurants in 18 states across USA that had posted nutrition information of their menu items on their website for 6 or more years | Calorie labelling laws – regulations requiring chain restaurants to post calorie information on menus and menu boards | Provide information | NR | Environmental restructuring; Education | Communication/marketing;  Environmental/social planning;  Legislation |
| Nothwehr 2013 | Repeat cross-sectional | 4 non-chain owner-operated restaurants in separate small towns in rural Iowa; full menu, sit-down restaurants with typical Midwestern fare. Community-based.  USA | Placement of 4 x 6 inch plastic signs at each table listing options for making order 'more healthful'. Top line stated 'healthy menu options now offered here' followed by bulleted list of 7 suggestions that had been developed in a pilot test in a similar restaurant. Owners in the study were shown this list as initial suggestion as well as results from their baseline survey of customer preferences. Suggestions included [number of restaurants agreeing]: Low fat salad dressing [4], low fat milk [4], leave out one + high fat ingredients [4], meat or fish grilled/baked instead of fried [4], whole-wheat bread [4], toppings on side [4], smaller portions available [3 - concern on price reduction by owner], sugar free syrup [1] , low sodium seasoning [1], breakfast items a la carte [1], low-fat dessert [1]. Sign in entry or window 'Ask about our healthy menu options'. Press release to local newspaper that interviewed owner | Enable choice | Social cognitive theory | Environmental restructuring; Education | Communication/marketing;  Environmental/social planning |
| Pandya 2013 | Repeat cross-sectional | Two Latino family-owned restaurants, Kansas City, USA. Community-based. | Healthy Restaurant Intervention: placing small menu stickers for identified healthier food options on menus. This intervention was an enhanced version of the Healthy Restaurant Award (HR Award) Program and both restaurants were recipients of the HR award (any restaurant in zip code 66101 offering minimum 6 of 12 identified criteria was eligible to receive the award which consisted of large stickers placed on restaurant door to designate that they offered healthier food options).  A Healthier Food Option was identified when it met any one of the categories: a) reduced portions of solid fats (i.e., lard or butter); b) replaced with lower fats such as olive oil, served with no or lower-fat cheese or sour cream; c) reduced calories in a menu item (e.g., replaced meat with fish/seafood, substituted chicken for pork or beef, used a smaller portion size as compared to other items on the menu); d) reduced sugar (e.g., no or less added sugar); e) reduced refined grains (e.g., reduced portions of refined grains, replaced refined grains with whole grains such as flour tortillas with wheat tortillas, white rice with brown rice); f) prepared or served with fresh veggies, and g) reduced sodium (e.g., no or less added salt). The elements of the Healthy Restaurant Intervention were developed and strategies were tailored to the cultural context of Latino family-owned restaurants. | Enable choice | Participatory Research | Environmental/restructuring; Education | Communication/marketing;  Environmental/social planning |
| Pulos & Leng 2010 | Repeat cross-sectional | 6 full service locally owned (non-chain) restaurants; 'casual, midrange' restaurants, Pierce County, Washington, USA | Voluntary nutrient labelling: restaurants displayed calories, fat, sodium, and carbohydrates for every regular food item, i.e. 750/21/2300/45 was most common format. Restaurants provided health department with recipes for their 'regular' menu items. On receiving feedback, some restaurants changed recipes, removed or added main meals. Restaurants received publicity as an incentive to participate and customers received an incentive (a  coupon for future dining, a pen, or a pedometer) for completing the survey. | Provide information | NR | Environmental restructuring; Education | Communication/marketing;  Environmental/social planning |
| Reimann 2015 | Cohort | One major chain sandwich restaurant, USA | Customers ordering a full sized (12 inch) sandwich were offered a half sized (6 inch) sandwich and non-food incentive (either a $10, $50 or $100 lottery) for the same price ($5) | Guide choices (incentives) | No framework but the theoretical background is discussed | Incentives | ? |
| Saelens 2012 | Controlled before and after study (retrospective) | 50 Fast food chain restaurants, King County, Washington, USA, 49 control restaurants in Multnomah County | Mandatory menu labelling in King County. In King County, chain restaurants with 15 or more sites nationally were required to post calorie information on their menus or menu boards. Information on saturated fat, carbohydrate, and sodium is also required, but not necessarily on the menu or near point of purchase. | Provide information | NR | Environmental restructuring; Education | Communication/marketing;  Environmental/social planning;  Legislation |
| Shah 2014 | Controlled clinical trial | One moderately priced restaurant in USA, which specialised in ‘small plates’ and opened 4pm – midnight. | Customers were offered one of four menus, depending on the day of the week they went to dine in the restaurant; healthy label menu, surcharge on unhealthy food (sin tax) menu, a menu that contained both, and ‘usual’ (control) menu. | Unhealthy label:  Enable choice  Sin tax:  Guide choice (disincentives)  Unhealthy label + sin tax:  Guide choice through disincentives | NR | Unhealthy label:  Environmental restructuring; Education  Sin tax:  Coersion  Unhealthy label + sin tax:  Environmental restructuring; Education; Coercion | Unhealthy label:  Communication/marketing; Environmental/  social planning  Sin tax:  Fiscal  Unhealthy label + sin tax:  Communication/marketing; Environmental/  social planning;  fiscal |
| Tandon 2011 | Controlled before and after study (same participants) | Chain restaurants in Seattle/King County, Washington versus restaurants in San Diego County, California, USA | Mandatory menu-labelling regulation in Seattle/King County: chain restaurants with 15 or more sites nationally were required to post calorie information on their menus or menu boards.  Participants were enrolled and sent a $10 gift card to a study eligible restaurant chain that they had reported they visit with their child. Parents were instructed to go to the restaurant with their child before the date of labelling implementation, purchase typical meals for themselves and their child, and mail back the receipt. All families returned to the same restaurant chain post-labelling. | Provide information | NR | Environmental restructuring; Education | Communication/marketing;  Environmental/social planning;  Legislation |
| Wansink 2014 | Repeat cross-sectional | McDonald’s restaurants, Nation-wide, USA | Changes to child meal bundle: 1. small size fry was replaced with an even smaller “kid size” (approx. 100 kcal); 2. a 1.2 oz. package of apple slices was included with all meals and the low fat caramel dip was discontinued; 3. advertising in restaurant and television promotions included a half pint milk jug containing 1% white or fat-free chocolate milk, while only the 1% white was featured previously. All other beverage options, such as apple juice and fountain beverages, were offered as before. | Restrict choice | NR | Environmental restructuring | Environmental/social planning; Communication/marketing |
| Wiggers 2001 | Repeat cross-sectional plus subgroup cohort | licenced hotels, clubs and nightclubs; in Hunter Valley, NSW region of Australia | Phone-based **direct marketing strategy**; variety of free services offered to businesses including menu guidelines for the provision of healthy choices. The direct marketing strategy was implemented on 6 occasions to address changes in the readiness of premises to adopt initiatives. Prior to each telephone contact, a report was provided to each premise that included tailored feedback regarding the initiatives previously reported to have been adopted by the premise and the prevalence of adoption by their industry peers. The project was promoted in the media (television, radio, print) via press releases and subsequent television, radio, and newspaper reports and also within the industry. | Enable choice | Contemporary health promotion theory -  creating supportive environments undertaken in variety  of community settings | Education | Communication/marketing;  Environmental/social planning;  Service provision |

**Key:** F: female; N/A: not applicable; NR: not reported; NYC: New York City; OHFO: out of home food outlet
