# Supplementary material for: The impact of interventions to promote healthier ready‐to‐eat meals (to eat in, to take away or to be delivered) sold by specific food outlets open to the general public: a systematic review
Source: Obes Rev. 2016 Nov 29;18(2):227–46. doi: 10.1111/obr.12479 (PMC5244662; doi:10.1111/obr.12479)
Supplement: Supplementary file 6 — Supporting info item [file OBR-18-227-s006.docx]

**Table S6 Results**

| Study ID | Global quality rating | Implementation score | Summary outcomes |
| --- | --- | --- | --- |
| Acharya 2006 | Moderate | 6 | The social marketing campaign did have a significant, small, indirect and direct effect on consumer purchase decisions. The campaign directly increased the probability of a consumer purchasing a healthy menu item by 3.7% compared with controls. Also, holders of campaign coupons were 17% more likely to purchase healthy menu items. By improving consumer attitudes toward healthy menu items, the campaign indirectly increased purchases of these items by 4.4%.  Consumers, who were more favourably predisposed toward healthy menu items, as measured by their attitudes, were more likely to purchase these items. A one-unit increase in the attitude index increased the probability of purchasing a healthy menu item by 4.4%. Moreover, consumers in the experimental market were 3.7% more likely to purchase the healthy menu items than those in the control region. These results show that the social marketing campaign did have a significant, albeit small, indirect and direct effect on consumer purchase decisions. The campaign coupons were also found to have played a significant role in influencing consumer purchases, as coupon holders were 17% more likely to purchase healthy menu items. Similarly, those who view nutrition as a primary factor influencing their purchase decision were more inclined to purchase the healthy menu items. Diners at certain restaurants also appeared to be more inclined to purchase the healthy menu items.  Diners’ perception toward healthy restaurant meals changed significantly during the campaign period. In particular, the postintervention measures indicate that consumers in the experimental region were more inclined to agree *(compared with controls)* with the statement, “Healthy menu items are tasty,” on a 5-point Likert-type scale ranging from *strongly disagree* (1) to *strongly agree* (5); the mean score in the experimental market was 3.33, whereas it was 3.23 in the control market. This difference in mean score is statistically significant at the 10% level (*p* = .10). Moreover, diners’ view on healthful meals improved in the intervention region during the course of the campaign, increasing from 3.18 during the preintervention period to 3.33 during postintervention. These changes in view are also significant from the baseline period at the 5% level (*t*-value = 2.74). |
| Angell 2012 | Moderate | 5 | Mean trans-fat content per purchase decreased by 2.4 g (2.9 vs. 0.5 g; 95% CI 2.8 to 2.0 g. Mean saturated fat content per purchase increased by 0.6 g (CI, 0.1 to 1.0 g. Mean trans-fat per 1000 kcal significantly decreased by 2.7 g per 1000 kcal. Purchases with zero grams of trans fat increased from 32% to 59%. Mean trans plus saturated fat content significantly decreased by 1.9 g overall. Results did not differ according to the poverty rate of the neighbourhood in which the restaurant was located. |
| Bagwell 2014 | Weak | 2 | Many of the criteria were already being met by the majority of the 77 businesses surveyed and only a small number of changes (average of 2.56) were needed to secure the HCC award. As might be expected, hot-food takeaway outlets had to make more changes than restaurants, but still only an average of 3.1 compared to 1.95 for restaurants. On the whole therefore, the businesses surveyed found the scheme relatively easy to adopt. |
| Bedard & Kuhn 2013 | Weak | 4 | The average transaction at the treatment store in the pre-treatment period included 1657 calories and 80 grams of fat. In comparison, the non-treated stores sold 3.7 (4.4) percent more calories (fat) per transaction.  In the post-treatment period, average calories and fat fell by about 2 percent in both treatment and control stores. No significant change in total calories per transaction; or fat of purchased items; purchases of 'encouraged' items increased. Intervention appears to be associated with a 2.7 percent reduction in cholesterol per transaction and a small increase in store revenue. |
| Bollinger 2011 | Strong | 5 | Food calories reduced by 14% on average (about 14 calories per transaction), but negligible impact on beverage calories. Average calories per transaction fall by 6 percent (from 247 calories to 232 calories per transaction). The effect is almost entirely related to changes in consumers’ food choices (small fraction of customers purchasing food)—there is almost no change in purchases of beverage calories. There is no impact on Starbucks profit on average, and for the subset of stores located close to their competitor Dunkin Donuts, the effect of calorie posting is actually to increase Starbucks revenue. 3/4 of the reduction in food calories due to consumers being less likely to purchase a food item, and 1/4 due to consumers substituting towards lower calorie food items. Cardholder individuals who averaged more than 250 calories per transaction prior to calorie posting reacted to calorie posting by decreasing calories per transaction by 26 percent. Survey results suggest that customers are less concerned about calories than taste and price. |
| Bruemmer 2012 | Weak | 3 | The paired analysis for main meals available at both time points indicated a significant decrease in energy when analysed for all chains (-41 +/- 156, p<0.0001). Saturated fat and sodium levels also decreased significantly across all chains and sit down chains (-0.7 +/- 3.8 g and -108 +/- 541 g respectively, both p<0.0001). Analysis of change between 6 months and 18 months in distribution of energy and nutrients for main meals available at either time point found significant shifts from higher quartiles to lower quartiles at sit down chains for energy, saturated fat, and sodium. No shift for energy, saturated fat, or sodium across quartiles for fast food chains overall or for fast food subtypes. Of main meals not designated for children, there was a slight shift between the proportion exceeding one third of the adult daily nutrient guidelines between 6 months and 18 months. The proportion of main meals that exceeded the recommended energy decreased from 60% to 56%, saturated fat 79% to 77%, and sodium 91% to 89%. |
| Chen 2015 | Weak | 5 | Calorie information awareness and use increased significantly from 2008 to 2010. Unadjusted analysis indicated that the proportion who saw and used calorie information rose from 8.1% to 24.8% over the two years; adjusted analysis confirmed significant increases. The largest absolute increases were among White, older, higher income, and more highly educated respondents. Cross sectional comparisons of subgroups after the policy went into effect identified a number of disparities; obese compared with ideal weight, and those eating at a fast food rather than a sit down chain restaurant, were more likely to see and use the calorie information. |
| Downs 2013 | Moderate | 4 | Calories purchased: NS interaction between calorie recommendations and calorie posting. Posting calorie recommendations had no direct impact, nor did it moderate the impact of calorie labels on food purchases. The recommendation appeared to promote a slight increase in calorie intake, attributable to increased purchases of higher-calorie main meals. |
| Dumanovsky 2011 | Moderate | 5 | Overall NS change in mean calories purchased (828 v 846 kcal; p=0.22); Sig reductions in three major chains (McDonalds, Au Bon Pain and KFC); Sig increase in Subway; 15% customers reported using calorie information and these customers purchased 106 fewer kilocalories than customers who did not see or use the calorie information (757 *v* 863 kcal). |
| Elbel 2009 | Moderate | 4 | 349 children/adolescents purchased 645 calories; 1156 adults purchased 846 calories after labelling.  Mandatory labelling associated with increase in noticing labelling but no significant change in calories purchased, or sat fat, sodium, sugar. No difference before and after labelling or between intervention and control sites, or by sex, race or age, for adults, adolescents, parents of children.  Although adolescents did appear to notice labels at similar rates to adults (57% vs 54%), those who did notice the information reported responding to it at somewhat lower rates than adults (16% vs 28%). Study was not powered to find a very small change in the amount of calories in youth.  Labelling didn’t change % participants who correctly estimated number of calories adult should consume daily (one third participants), but labelling did increase % who could accurately estimate calories in their purchase. Change in the % correctly estimating before/after the intervention in the intervention area (from 15% before to 24% after in NYC), suggesting that calorie labelling may improve knowledge. However, overall knowledge and correct estimation of calorie content of fast food remained very low. Adults in NYC who reported noticing and using the calorie labels consumed fast food less frequently compared to adults who did not notice the labels (4.9 vs. 6.6 meals per week). Purchase of high Calorie beverages decreased in the control city and increased in the intervention city before/after implementation (P<0.05 for difference in differences (DD). Purchase of regular (high fat) salad dressing increased in the intervention city post intervention and decreased in the control city post intervention (DD p<0.01). In NYC (intervention city only) those who saw calorie information and said they had acted on it were 15% more likely to buy a salad post-intervention Implementation (p<0.05). Those who saw and acted on calorie info were also less likely to buy a fast food lunch (0.64 fewer), dinner (0.48 fewer) and total fast food purchases per week than those who did not notice calorie information. No other changes significant. |
| Elbel 2013 | Moderate | 5 | The difference in number of calories purchased as assessed from purchase receipts pre and post legislation (2009-2010) was not significant in Philadelphia (legislation city) or Baltimore (no legislation). Mean (sd) number of calories purchased in Philadelphia pre 959, post 904 (change -55, p=0.167); Baltimore pre 992, post 940 (change -52, p=0.276). |
| Eldridge 1997 | Weak | 6 | Low fat sandwiches increased (18.5% to 21.7% of total sandwich sales at final quarter; p<0.05); low-fat snacks increased (10.5% to 14.9% of total snack sales; p<0.05); cheese pizza (7.7% - 25.1%, p<0.05); salads and frozen yogurts were unaffected by intervention. |
| Finkelstein 2011 | Moderate | 3 | Neither total monthly transactions nor calories per transaction were affected immediately by the legislation or affected later when calorie information was added to the drive-through menu boards. Average calories per transaction were roughly 180 calories greater in the non–King County, compared with the King County locations, both before and after the menu-labelling law went into effect; King County patrons already making healthier purchases before the law took effect. Lower-calorie food options were identifıed as the Healthy Highlights listed on the company’s menu and website prior to the intervention. |
| Fitzgerald 2004 | Weak | 6 | The percentage of heart-healthy items sold after the campaign showed a trend toward a slight increase in heart-healthy menu item selections, although it was not statistically significant. 4 of 9 restaurants showed small/no increase in % (range 0-25% increase) of HDP menu items sold; 5 restaurants showed decrease (-2 to -9%). Throughout HDP items accounted for 28-33% of sales. |
| Gase 2015 | Weak | 6 | Differences between the pre and post-menus were noted for 12 of the 17 brands; the remaining 5 brands qualified for program participation without the need for any menu changes. Nine of the 17 brands made changes to their menus to meet the first program participation criterion (offering reduced-size portions). The remaining 8 had menus that already complied with program participation criteria. Three restaurants increased the number of reduced size menu items by 1 to 25 percentage points, while 6 increased the number of reduced-size menu items by 26 to 50 percentage points. Three restaurants increased the number of sections in which reduced-size options were available by 1 to 25 percentage points and 6 increased the number of sections by 26 to 50 percentage points. The three most frequently offered options available in reduced-size portions (in terms of percentage of menu items offered) were salads, sandwiches or burgers, and pasta, pizza, or grain-based entrées. On the post participation menus, the average price of reduced-size item was $7.13 (SD = 3.17), compared with $10.72 (SD = 4.32) for the corresponding regular size item.  Ten of the 17 restaurant brands offered children’s meals; 8 of the 10 made changes to their children’s meals (the remaining 2 had menus that already complied with program participation criteria). Six changed the type of beverages they included with children’s meals from those considered “less healthy” to those considered “healthy” based on program criteria (5 restaurants) or from those considered “less healthy” to none (1 restaurant). Seven changed their meal offerings to include at least one non-fried fruit or vegetable as the default meal option, while 6 decreased the number of meals containing fried foods. |
| Hanni 2009 | Weak | 9 | Of the 16 taquerias contacted by outreach efforts from July 2006 through August 2007, 94% of owners agreed to and began distributing health education materials on healthy lifestyle choices, such as eating healthy and exercising. 81% introduced or began promoting one or healthier side options (whole beans, whole wheat tortillas, and/or freshly made fruit juice). 50% began promoting entrées that were based on healthier food options; of these, seven (88%) modified existing entrées or created entrées that were healthier (chicken or vegetable burritos, whole beans with vegetables, salad entrée, or a grilled fish entrée). |
| Horgen & Brownell 2002 | Weak | 6 | Overall, the price decrease and combination interventions produced the strongest increase in the target items. Sales of target items rose during each intervention period and decreased when each intervention was removed. Average daily sales of all items were not significantly different by period. Target and control items reacted differently across period - 39% variability in target item sales due to period; 6% for control items. The framing manipulation of the messages had no significant effect on sales. Price decreases alone may be more effective than a combination of price decreases and health information for increasing sales of some foods. Price decreases alone were more effective in promoting sales of the chicken sandwich and chicken salad but the combination intervention was more effective for the soup. |
| Krieger 2013 | Moderate | 4 | Mean calories per purchase decreased 18 months after implementation of menu labelling in some restaurant chains and among women but not men. NS changes in calories purchased between BL and P1 in either food or coffee chains. Unadjusted mean calories decreased from BL to P2 by 38 kcal in food chains (p=0.06, 95% CI= -76.9, 0.8) & 22 kcal in coffee chains (p=0.002, 95% CI = -35.8, -8.5). No significant changes occurred between baseline and 4–6 months post-regulation. Mean calories per purchase decreased from 908.5 to 870.4 at 18 months post- implementation in food chains and from 154.3 to 132.1 in coffee chains.  Calories decreased in taco and coffee chains, but not in burger and sandwich establishments. They decreased more among women than men in coffee chains. Awareness of labels increased from 18.8% to 61.7% in food chains and from 4.4% to 30.0% in coffee chains (both p=0.001). Among customers seeing calorie information, the proportion using it (about one third) did not change substantially over time. After implementation, food chain customers using information purchased on average fewer calories compared to those seeing but not using (difference <143.2 kcal, p=0.001) and those not seeing (difference<135.5 kcal, p=0.001) such information. |
| Lee-Kwan 2013 | Moderate | 8 | Sales of healthy menu items significantly increased across all intervention carryouts, while foods that were not promoted either decreased or stayed the same.  100% seen menu board. 84.2% seen menu labels. 34.7-55.4% seen each of 3 posters. 42.5% purchased logo-promoted food. 65.3% purchased photo-promoted. Reach: 36.8% increase in promoted dishes sold in intervention vs 1.2% increase in control stores. There was a strong dose response relationship between the combined intervention exposure score (range 0–24) and the frequency of healthy food purchased (beta 0.38, CI 0.26–0.49). Among customers exposed to the intervention, there were increased odds of purchasing specific healthy food items, such as a turkey club sandwich (OR 1.54, p<0.0001), fruit cup (OR 1.37, p<0.0001), or cooked greens (OR 1.30, p<0.0001). |
| Licata 2002 | Weak | 7 | There was no significant change in the percentage of restaurants and cafes undertaking nutrition-related health promotion practices between 1997 and 2000. Cohort results: n=122; 105 (86%) undertaking Nutrition HPP in 1997; 102 (84%) in 2000; no sig change (p=0.66); Cross-sectional results: 268 (83%) in 1997 (total n=321); 198 (83%) in 2000 (total n=239); no sig change (p value NR). Exposure: 87 (71%) had been exposed to two telephone marketing calls; 35 (29%) had been exposed to three calls. For both cross-sectional and cohort samples, there was a change in at least one area with the exception of nutrition. ‘Nutrition’ was the only health area with only one health promotion initiative; all the other health areas had more than one initiative. |
| Namba 2013 | Strong | 3 | No change in average calorie content of main meals and sides in either control or case restaurants (and thus no difference in change). Case-by-case analysis highlighted the heterogeneity in restaurant trends: 3 of 5 cases improved menu offerings, while 2 of 5 showed no improvement and even launched new options that increased average calories by almost 20% and cholesterol by almost 140%. However, the % of healthier items listed on the menus increased in case (from 13% to 20%) but not control (remained at 8%) restaurants (test for difference in the trend p=0.02). NS changes over time of adult menu nutrient averages and NS between cases and controls; 3 of 5 cases improved menu offerings and 2/5 no improvement/worsened;  Children’s menu items – When healthier options were defined as 3 or more out of 5 nutritional criteria, the proportion of healthier children’s menu choices at case restaurants was much higher than at control restaurants. No difference was observed in trend over time: prevalence of meeting 3 of 5 criteria was around 60% for cases and 10% for controls (difference in prevalence, n = 22, p=0.02; test for interaction in regression trend analysis, p=0.6). |
| Nothwehr 2013 | Weak | 8 | Over 12-months there was no significant trend in the mean composite scores of healthy choices which were assessed using order slips. Order slip analysis: mean composite score estimates of healthy choices = 1.13, 1.07, 1.09 and 1.05 (baseline-T4) - no significant time trend (p=0.37).  A brief survey showed that the percentage of customers eating out at least three times a week significantly decreased over time (attributed to changing economy). There was no statistically significant difference across time points in the proportion who noticed the window signs, noticed the table signs, or stated that the signs affected their order somewhat or a lot; 34% of customers that reported noticing reported that the signs affected their order. Approximately 40 to 58% noticed the window signs and 67 to 71% noticed the table signs. Interviews with owners and waiting staff indicated study procedures and programme not disruptive and customers were appreciative of the programme and asked more questions about food content and preparation than prior to the programme. |
| Pandya 2013 | Weak | 7 | There was no significant improvement in customers’ purchasing behaviours of healthier main meals or regarding individual healthier options. 6.6% (of 600 receipts) to 3.95% (of 1193) in one restaurant; 3.22% (of 140) to 4.31% (of 236) in the other. For Restaurant 1, a total of 28 modifications were made in nine different menu items. For Restaurant 2, a total of 18 modifications were made in nine different menu items. This represented an average of 2-3 modifications per menu item. |
| Pulos & Leng 2010 | Weak |  | On average, over all restaurants, significant decreases in Calories, Fat and Sodium, pre to post-labelling. No difference in Carbohydrates. The average post-labelling main meal sold contained about 15 fewer calories, 1.5 fewer grams of fat, and 45 fewer milligrams of sodium than did the average main meal sold before labelling. 71% patrons reported noticing the nutrition information; 20.4% reported ordering an main meal lower in calories as a result, and 16.5% reported ordering an main meal lower in fat. |
| Reimann 2015 | Moderate | 7 | 5%, 8% and 22% of customers changed their menu choice from a full sized sandwich to a half sized sandwich with the offer of a $10, $50 and $100 lottery, respectively. There was a significant negative effect of the magnitude of the non-food incentive on full sized portion choice (regression model *B* = -0.01, SE = 0.01, Wald = 4.28, P = 0.039). Women chose the full-sized portion comparatively more often than men (96% vs 88%; *B* = 1.16, SE = 0.44, Wald = 6.92, P = 0.039). Age, BMI and hunger level did not appear influence the effect of the magnitude of non-food incentive on portion size choice. |
| Saelens 2012 | Strong | 4 | Adults: There were significant differences at Wave 1 between King and Multnomah County restaurants in facilitators of healthy eating (with and without nutrition labelling items) and barriers to healthful eating. Overall availability of healthy options and facilitation of healthy eating did not differentially increase in King County versus Multnomah County restaurants. Mixed findings but some positive changes to environment (decrease in encouragement to overeat or eat unhealthily) in intervention vs control counties, but no increase in identifying or providing more-healthful options.  Children: There was a significant improvement in facilitators of healthy eating and the healthfulness of the kid’s menu in King relative to Multnomah County restaurants, although this differential change by county no longer remained once nutrition-labelling items were removed from these NEMS-R scales. There was a significant improvement in the healthfulness of the kid’s menu, particularly from before to the most distal post-regulation time point (Wave 3), even after removing nutrition-labelling items from the Kid’s Menu scale, but not differentially by county. |
| Shah 2014 | Strong | 5 | The difference in the mean proportion of unhealthy main dishes (entrees) ordered per table (table = unit of analysis) varied significantly between the four menu conditions; 50% no intervention; 46% surcharge (sin tax); 30% unhealthy label; 29% surcharge + unhealthy label. Adding a surcharge made no significant difference, but adding an unhealthy label or a surcharge + unhealthy label did. However, the effect of the interventions was different in men compared with women. The proportion of unhealthy meals ordered per table in the control (no intervention) group was slightly higher (but not sig) for ‘female tables’ (defined as tables where the majority of people at that table were female) compared with male tables. The proportion of unhealthy meals ordered per table in the surcharge group was slightly lower (but not sig) for female tables compared with male tables. The proportion of unhealthy meals ordered per table in the unhealthy label group and the surcharge + unhealthy label group was significantly higher for male tables compared with female tables (60% vs 17%; 36% vs 25%, respectively). |
| Tandon 2011 | Weak | 4 | Restaurant menu-labelling regulation increased parent's nutrition information awareness, but did not decrease calories purchased for either children or parents.  In S/KC, there was a significant increase from pre- to post-regulation (44% vs 87%) in parents seeing nutrition information, with no change in SDC (40% vs 34%). Average calories purchased for children did not change in either county (823 vs 822 in S/KC; 984 vs 949 in SDC).  There was an approximately 100-calorie decrease for the parents postregulation in both counties  (823 vs 720 in S/KC; 895 vs 789 in SDC), but no difference between counties. Parents decreased calories by approximately 100 from pre- to post-regulation in both counties regardless of whether or not they saw nutrition information in unadjusted and adjusted models. Calorie data were adjusted for parent's gender, race and household income. Analyses stratified by child gender and weight status (child overweight/obese BMI ≥85th %; parent overweight/obese BMI ≥25) were conducted. |
| Wansink 2014 | Weak | 3 | Side items (apples and French fries) served in the average “4-item” meal purchased in 2012 had 98 fewer kcal (P<0.001) compared with “3-item” meals purchased in 2011, accounting for 94% of the 104-kcal decrease for the entire meal (P<0.001). There did not appear to be a compensatory effect of the portion and calorie reduction, in terms of the entrée selection. There was no change in the percentage of customers selecting the chicken nuggets (the entrée with the lowest energy value [190kcal]), however there was a small decrease in hamburger (250 kcal) selections (11.1% to 10.1%; P<0.05) and a small increase in cheeseburger (300 kcal) selections (28.3% to 29.3%; P<0.01).  Selection of regular caloric soda (as part of the child meal bundle) decreased (from 58.1% to 51.8%; P<0.001) while chocolate milk selections increased (from 16.5% to 20.3%; P<0.001). |
| Wiggers 2001 | Weak | 7 | Marked and statistically significant increases in the prevalence of reported Healthy eating initiatives in licensed clubs, nightclubs and hotels serving food. Of 77 outlets serving food, 42% reported serving healthier food options at baseline, and 96% at follow up (cohort data).Cross sectional: 77 (35%) to 99 (97%) (p<0.001) |

**Key:** F: female; N/A: not applicable; NR: not reported; NYC: New York City; * Data taken from figures and, therefore, estimated values
